# CHAC1: a master regulator of oxidative stress and ferroptosis in human diseases and cancers

**DOI:** 10.3389/fcell.2024.1458716

**Published:** 2024-10-29

**Authors:** Jiasen Sun, Hui Ren, Jiawen Wang, Xiang Xiao, Lin Zhu, Yanyan Wang, Lili Yang

**Affiliations:** ^1^ Department of Gastroenterology, Ankang Central Hospital, Ankang, Shaanxi, China; ^2^ Department of Cardiovascular Disease, Ankang Central Hospital, Ankang, Shaanxi, China

**Keywords:** ferroptosis, cell death, unfold protein response, endoplasmic reticulum stress, cancer, glutathione

## Abstract

CHAC1, an essential regulator of oxidative stress and ferroptosis, is increasingly recognized for its significant roles in these cellular processes and its impact on various human diseases and cancers. This review aims to provide a comprehensive overview of CHAC1’s molecular functions, regulatory mechanisms, and effects in different pathological contexts. Specifically, the study objectives are to elucidate the biochemical pathways involving CHAC1, explore its regulatory network, and discuss its implications in disease progression and potential therapeutic strategies. As a γ-glutamyl cyclotransferase, CHAC1 degrades glutathione, affecting calcium signaling and mitochondrial function. Its regulation involves transcription factors like ATF4 and ATF3, which control CHAC1 mRNA expression. CHAC1 is crucial for maintaining redox balance and regulating cell death pathways in cancer. Its elevated levels are associated with poor prognosis in many cancers, indicating its potential as a biomarker and therapeutic target. Additionally, CHAC1 influences non-cancerous diseases such as neurodegenerative and cardiovascular disorders. Therapeutically, targeting CHAC1 could increase cancer cell sensitivity to ferroptosis, aiding in overcoming resistance to standard treatments. This review compiles current knowledge and recent discoveries, emphasizing CHAC1’s vital role in human diseases and its potential in diagnostic and therapeutic applications.

## 1 Introduction

Oxidative stress and ferroptosis are critical cellular processes with significant implications for various human diseases and cancers. Oxidative stress refers to an imbalance between the production of reactive oxygen species (ROS) and the capability of the cell to detoxify these reactive intermediates or repair the resulting damage. This imbalance can lead to cellular damage and has been linked to numerous pathological conditions, including neurodegenerative diseases, cardiovascular disorders, and cancers (Forman and Zhang, 2021). Ferroptosis, on the other hand, is a distinct form of regulated cell death characterized by iron-dependent accumulation of lipid peroxides (Chen L. et al., 2023; Tabnak et al., 2021a). Unlike apoptosis or necrosis, ferroptosis is driven by the loss of activity of glutathione peroxidase 4 (GPX4), an enzyme that protects cells from lipid peroxidation. The initiation of ferroptosis involves the depletion of GSH, an essential cellular antioxidant, which leads to the accumulation of toxic lipid ROS and eventual cell death (Tang et al., 2021). CHAC glutathione-specific gamma-glutamylcyclotransferase 1 (CHAC1) has been identified as a crucial factor in maintaining redox balance and regulating cell death pathways. It functions as a γ-glutamyl cyclotransferase, explicitly acting on glutathione, thereby influencing calcium signaling and mitochondrial respiratory function (Kumar et al., 2012). The regulation of CHAC1 is complex, involving transcriptional and post-transcriptional mechanisms. Activating transcription factors such as ATF4 and ATF3, along with a bipartite ATF/CRE regulatory element, have been shown to control CHAC1 mRNA induction. Recent research has highlighted the role of ChaC1 in cancer progression (Zhang et al., 2024). For example, MIA3 has been shown to promote the degradation of GSH by binding to CHAC1, thus facilitating hepatocellular carcinoma progression (Wanbiao et al., 2023). Additionally, TRIB3 enhances cell resistance to arsenite toxicity by limiting the expression of CHAC1, indicating a protective role in certain stress conditions (Örd et al., 2016). In the context of ferroptosis, ChaC1 degradation of GSH enhances susceptibility to ferroptosis under cystine starvation conditions (Dixon et al., 2014). ChaC1’s involvement extends beyond cancer, impacting other diseases and cellular processes. For instance, its activity is crucial in spinal cord injury recovery, where CD36 deletion has been shown to improve outcomes. ChaC1 has been implicated in the unfolded protein response (UPR) in oxidative stress, acting downstream of the ATF4-ATF3-CHOP cascade (Crawford et al., 2015). Furthermore, its role in ferroptosis has therapeutic implications, as evidenced by studies demonstrating that pharmacological inhibition of cystine-glutamate exchange induces ferroptosis in various cancer cells, presenting a potential avenue for cancer treatment (Wang et al., 2019). This review aims to provide a comprehensive overview of ChaC1, focusing on its molecular functions, regulatory mechanisms, and its dual role in oxidative stress and ferroptosis. We will explore the biochemical pathways involving ChaC1, explore its regulatory network, and discuss the implications of its activity in different pathological contexts. By synthesizing current knowledge and recent findings, this review seeks to elucidate the critical contributions of ChaC1 to human diseases and cancers, highlighting its potential as a biomarker and therapeutic target.

## 2 Overview of CHAC proteins

The CHAC1 gene is located on chromosome 15q15.1 and codes for an enzyme involved in the breakdown of GSH, a critical cellular antioxidant (Yao and Sherif, 2016). This enzyme, γ-glutamylcyclotransferase, breaks down GSH into cysteine-glycine and 5-oxoproline, which plays a significant role in managing oxidative stress within cells (Kumar et al., 2015). CHAC1 expression is triggered by oxidative stress, particularly through the UPR pathway during endoplasmic reticulum (ER) stress (Mungrue et al., 2009). As a pro-apoptotic molecule, CHAC1 influences several forms of programmed cell death, including apoptosis and ferroptosis, making it vital in how cells respond to stress (Zhou and Zhang, 2023). In cancer, CHAC1’s function varies depending on the tumor type. It can promote cell death by reducing intracellular GSH levels, although in some cases, it may contribute to cancer progression depending on its interactions with stress-related cellular mechanisms (Zhang et al., 2024). However, the *CHAC2* gene is located on chromosome 2p16.2 (Chand et al., 2022). Unlike CHAC1, which is induced by stress, CHAC2 is consistently expressed under normal conditions and has a lower capacity for GSH degradation. CHAC2 primarily regulates baseline GSH turnover, helping to maintain cellular redox balance without inducing excessive stress (Chand et al., 2022; Wang et al., 2017; Liu et al., 2017; Kaur and Bachhawat, 2016; Kaur et al., 2017). This protein plays an essential role in normal development, especially by supporting the self-renewal and pluripotency of human embryonic stem cells (hESCs). A reduction in CHAC2 expression disrupts the cell cycle and leads to cell death, highlighting its importance in early development processes (Wang et al., 2017). In disease contexts, CHAC2 is often found at reduced levels in some cancers, such as gastric and colorectal cancers, where it acts as a tumor suppressor by promoting apoptosis and autophagy through ER stress pathways. However, in certain cancers like breast cancer, CHAC2 expression may be elevated, contributing to tumor progression by increasing oxidative stress and activating pathways such as MAPK signaling. This suggests that CHAC2 plays a dual role, maintaining cellular balance in normal conditions while influencing cancer development in a context-specific manner (Reviewed in (Zhang et al., 2024)).

## 3 Molecular functions of ChaC1

ChaC1 and its homologues function as γ-glutamyl cyclotransferases (GCTs) that specifically degrade glutathione. Structural analysis shows they share a fold similar to other GCTs. *In vivo* yeast assays demonstrated that ChaC1 and its homologues promote growth on GSH by breaking it down into 5-oxoproline and cys-gly, a function lost in catalytic mutants. *In vitro* assays confirmed their specific activity towards glutathione, with relevant kinetic parameters. Overexpression of ChaC1 in yeast caused significant GSH depletion and increased apoptosis, which could be reversed by adding glutathione, linking ChaC1’s role in GSH degradation to apoptosis (Kumar et al., 2012). ChaC1 is expressed early in zebrafish development, particularly in the muscles, brain, and heart. When ChaC1 is knocked down, severe developmental defects and embryonic lethality occur. These effects can be reversed by introducing wild-type ChaC1 but not by a catalytically inactive mutant, emphasizing the importance of its enzymatic function. Grx1-roGFP2 was used to measure intracellular GSH redox potential, showing that ChaC1 knockdowns had a less oxidizing environment than wild-type zebrafish. This alteration in redox potential affected calcium signaling, as calcium transients, observed using GCaMP6s, were significantly reduced in the knockdowns. The degradation of GSH by ChaC1 and the resulting shift in redox potential are key upstream factors that activate calcium signaling, which is essential for the proper development of zebrafish muscles, brain, and heart (Yadav et al., 2019). Suyal et al. emphasized the importance of specific ChaC1-exclusive residues in maintaining the enzyme’s structural and functional stability. Through sequence alignment and structural analysis, they identified a unique conserved motif, “RRFQWQTHRGPGR,” in the ChaC family. Mutational analysis revealed key residues (R63, F64, W65, H71, R72, G73, R80) crucial for enzyme function, despite not directly binding the substrate. These residues stabilize the active site, ensuring proper substrate positioning. Mutations in these residues disrupted the enzyme’s structure, leading to reduced stability and lower substrate binding affinity, underscoring their critical role in ChaC1’s function (Suyal et al., 2023). ChaC1 has three isoforms: Isoform A, Isoform B, and Isoform X1, which vary in their structure and functionality. Isoform A, the primary form, consists of 222 amino acids and plays a key role in breaking down cytosolic glutathione, essential for maintaining redox balance and supplying amino acids like cysteine and glycine. Isoform B, which is shorter by 44 amino acids, is inactive, while Isoform X1, a longer form with 264 amino acids, is not found in some higher species (Parande et al., 2024).

### 3.1 Molecular interactions of CHAC1

**FIGURE 1:**
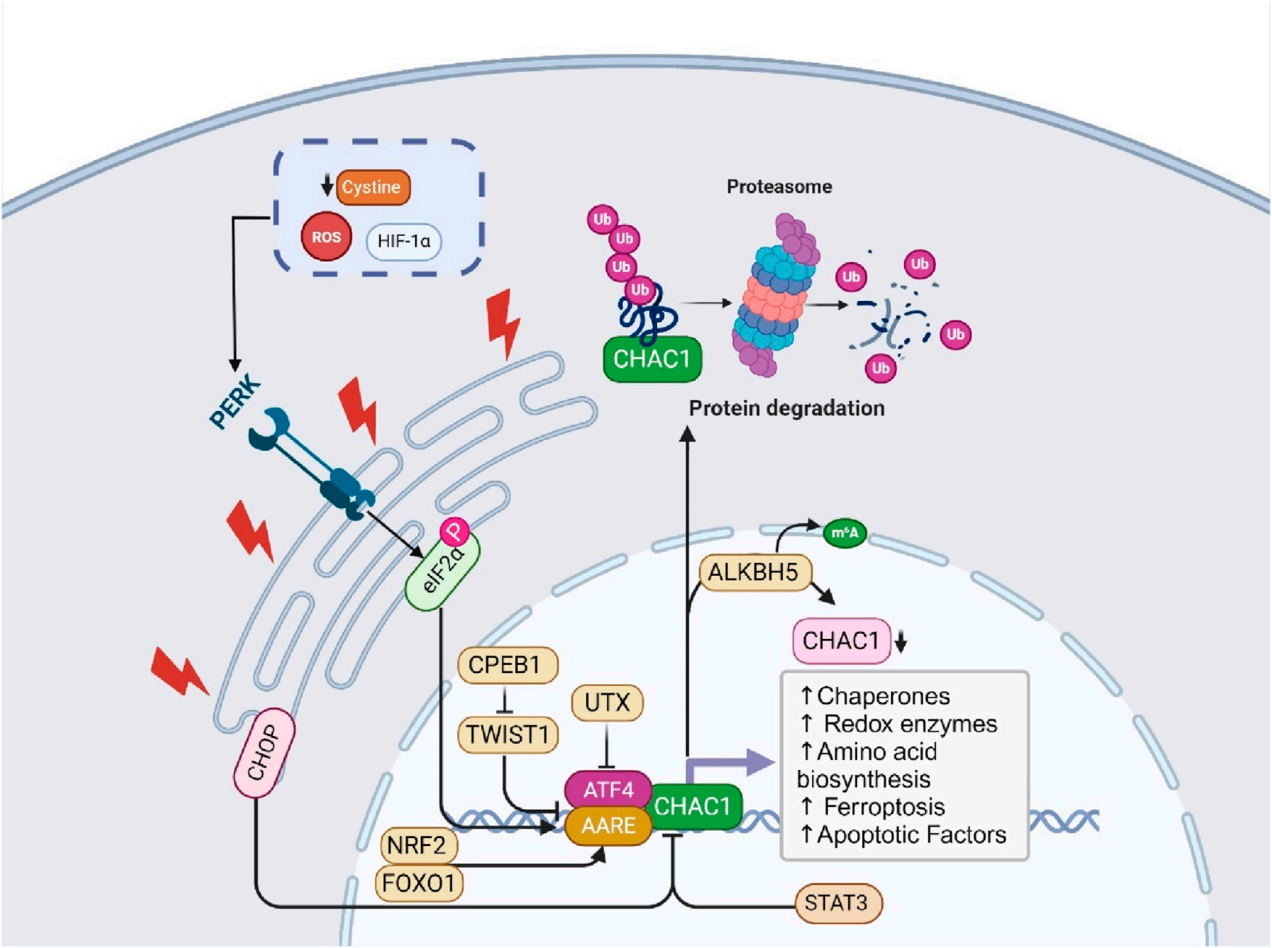
This figure illustrates the molecular functions and regulatory mechanisms of ChaC1. ChaC1 acts as a γ-glutamyl cyclotransferase, involved in glutathione degradation, producing 5-oxoproline and cysteinylglycine. This process leads to cellular GSHdepletion and increased apoptosis. The regulation of ChaC1 is multifaceted, involving several pathways. Under ER stress, the PERK-eIF2α-ATF4 pathway is activated, leading to the transcriptional upregulation of ChaC1. Additionally, CHOP, another stress-induced factor, inhibits ChaC1 transcription indirectly. Post-transcriptional and epigenetic regulation also plays a significant role, where ALKBH5-mediated m6A demethylation reduces ChaC1 mRNA stability, and the histone demethylase UTX suppresses ATF4, indirectly regulating ChaC1 expression. Furthermore, ChaC1-induced GSHdepletion activates the NRF2 pathway, enhancing antioxidant defense mechanisms. Other regulatory factors include STAT3, which negatively regulates ChaC1, and FOXO1, which positively regulates ChaC1 through coordinated action with C/EBPδ and ATF4. These intricate regulatory mechanisms highlight the complex role of ChaC1 in cellular responses to stress, apoptosis, and redox homeostasis.

The regulation of CHAC1 is multifaceted, involving various cellular stress and damage response pathways. Primarily, CHAC1 is transcriptionally regulated by ATF4, a key player in the integrated stress response (ISR), which is activated during conditions like amino acid deprivation and oxidative stress (Crawford et al., 2015). Additionally, CHAC1 is upregulated through the PERK-ATF4 pathway during ER stress (Xu Y. et al., 2022) and can be influenced by the tumor suppressor protein p53 in response to DNA damage (Wada et al., 2018). It also responds to oxidative stress and hypoxic conditions, common in solid tumors, through mechanisms involving ROS and hypoxia-inducible factors (HIFs) (Kihira et al., 2023). Nutrient deprivation, particularly cystine and amino acid starvation triggers CHAC1 expression to help the cell adapt (Chen M.-S. et al., 2017). While less characterized, post-translational modifications may further regulate CHAC1’s stability and activity (Chen C. et al., 2023). This diverse regulatory network allows CHAC1 to play a crucial role in maintaining cellular homeostasis and adapting to environmental stresses. In this section, we discuss the detailed molecular regulation of ChaC1 (Figure 1).

#### 3.1.1 Transcriptional and post-translational regulation

Chac1 is a novel pro-apoptotic factor induced by ER stress. ChaC1 mRNA expression is upregulated in response to ER stress-inducing agents such as thapsigargin, tunicamycin, and brefeldin A. The transcriptional regulation of ChaC1 involves two critical elements in its promoter: the ATF4 and the amino acid response element (AARE). Both components are essential for basal promoter activity and responsiveness to ER stress, and mutations in these elements significantly decrease promoter activity. Post-translationally, ChaC1 protein is highly unstable and is rapidly degraded via the proteasome pathway, as its expression could only be detected when the cells were treated with the proteasome inhibitor MG132. This dual regulation highlights the intricate control of Chac1 expression, linking its transcriptional activation by stress signals to its proteasomal degradation, which is crucial for maintaining cellular homeostasis under stress conditions (Oh-hashi et al., 2013).

##### 3.1.1.1 Ubiquitination

Ubiquitination plays a complex role in regulating the stability and function of the Chac1 protein. ChaC1 is frequently ubiquitinated, generally tagging proteins for degradation via the proteasome pathway. However, intriguingly, the ubiquitination of ChaC1 does not always lead to its degradation. For instance, ChaC1 was stabilized when co-transfected with ubiquitin genes, suggesting that ubiquitination can also enhance ChaC1 stability under certain conditions. This dual role of ubiquitination in both potentially targeting ChaC1 for degradation and stabilizing it highlights a nuanced regulatory mechanism where the context and perhaps the nature of the ubiquitin linkages or chains may dictate the ultimate fate of the ChaC1 protein. Such findings suggest a sophisticated level of control over ChaC1’s role in cellular responses to stress, particularly in managing intracellular GSH levels (Nomura et al., 2016).

##### 3.1.1.2 ATF4 and ATF3

CHAC1, an enzyme that degrades intracellular glutathione, is a direct target of the transcription factor ATF4, activated by UPR signals via the PERK-EIF2A pathway. The researchers identified two conserved DNA elements in the CHAC1 promoter, ATF/CRE and CARE, which synergistically regulate CHAC1 transcription. Using luciferase reporter assays and DNA binding studies, they demonstrated that ATF4 directly binds to these elements, enhancing CHAC1 expression under ER stress conditions induced by chemicals like Thapsigargin and Tunicamycin. Interestingly, CHOP, another UPR-associated transcription factor, was found to inhibit CHAC1 transcription via the ATF/CRE element without directly binding to it. This suggests an indirect regulatory mechanism possibly involving interactions with other transcription factors, such as ATF4. The study concludes that CHAC1 is a novel UPR target gene negatively regulated by CHOP, revealing complex regulatory crosstalk in the UPR pathway and providing insights into the molecular mechanisms of oxidative stress and cell death (Nomura et al., 2020). The regulation of CHAC1 by ATF4 and ATF3 involves a complex interaction with specific DNA elements within the CHAC1 promoter region. Two critical cis-regulatory elements, the ATF/cAMP response element (ATF/CRE) located at −267 and a novel ATF/CRE modifier (ACM) element at −248, are necessary for the promoter-driven transcription of CHAC1 under ER stress conditions. Both ATF4 and ATF3 bind to these elements, with ATF4 binding primarily to the ATF/CRE site and the ACM site, especially under stress conditions, while ATF3 binds to the ATF/CRE site in both basal and stress conditions. Luciferase reporter assays demonstrated that mutations in these elements significantly diminished the promoter activity, confirming their essential role in CHAC1 transcription. Additionally, chromatin immunoprecipitation (ChIP) assays confirmed the enrichment of ATF4, ATF3, and CEBPβ at these sites in the CHAC1 promoter following ER stress. This interaction highlights the coordinated regulation where ATF4 and ATF3 binding to these elements modulates CHAC1 expression, linking it to the cellular stress response pathways (Crawford et al., 2015; Romanoski et al., 2011). UTX, a histone H3 lysine 27 (H3K27) demethylase, suppresses ATF4 by modulating the expression of genes involved in the integrated stress response (ISR). UTX functions as part of multiprotein complexes that regulate gene expression by removing repressive histone marks. UTX negatively regulates the expression of ATF4 and its downstream target genes, such as ChaC1, by maintaining histone marks that prevent their activation. In UTX-deficient cells, or when the KDM6 inhibitor GSK-J4 inhibits UTX activity, ATF4 and its target genes, including ChaC1, are upregulated. This induction is primarily mediated through the activation of the heme-regulated eIF2α kinase (HRI), which leads to increased ATF4 translation and the subsequent activation of genes involved in the stress response, highlighting UTX’s role in modulating cellular stress pathways and its potential impact on cancer progression (Kitajima et al., 2021).

##### 3.1.1.3 CPEB1

Cytoplasmic Polyadenylation Element Binding Protein 1 (CPEB1) is a post-transcriptional regulatory protein involved in mRNA translation. In gastric cancer (GC) cells, CPEB1 has been shown to enhance erastin-induced ferroptosis, a form of iron-dependent cell death. CPEB1 achieves this by downregulating the expression of TWIST1, a transcription factor that inhibits ATF4. By suppressing TWIST1, CPEB1 indirectly activates the ATF4/CHAC1 pathway. CHAC1, in turn, GSH, leading to increased levels of ROS and promoting ferroptosis in GC cells. Thus, CPEB1 regulates CHAC1 by modulating the TWIST1-ATF4 pathway, highlighting its role in ferroptosis and potential as a therapeutic target in cancer treatment (Wang J. et al., 2021).

##### 3.1.1.4 m^6^A modification

N6-methyladenosine (m^6^A) is the most prevalent internal modification of mRNA in eukaryotes, influencing various aspects of RNA metabolism, including stability, splicing, translation, and localization. This dynamic modification is installed by m6A methyltransferases (writers) and removed by demethylases (erasers) such as ALKBH5. In gastric cancer, ALKBH5-mediated m6A demethylation regulates the stability of target mRNAs, including CHAC1. By removing m^6^A marks from CHAC1 mRNA, ALKBH5 decreases its stability, leading to reduced CHAC1 expression. CHAC1, in turn, modulates intracellular ROS levels, impacting cell proliferation, metastasis, and chemotherapy sensitivity. Thus, m6A modification, through the action of ALKBH5, plays a crucial role in the regulation of CHAC1 and subsequent cancer cell behavior (Chen C. et al., 2023).

##### 3.1.1.5 NRF2

Nuclear factor erythroid 2–related factor 2 (NRF2) is a transcription factor that plays a pivotal role in the cellular defense against oxidative and electrophilic stress. It regulates the expression of various genes involved in antioxidant response, detoxification, and cellular metabolism. Under normal conditions, NRF2 is bound to its inhibitor, Kelch-like ECH-associated protein 1 (KEAP1), which facilitates its degradation through the ubiquitin-proteasome pathway. However, under stress conditions, modifications of KEAP1 disrupt this interaction, allowing NRF2 to accumulate and translocate into the nucleus. In the nucleus, NRF2 binds to antioxidant response elements (ARE) in the promoter regions of its target genes, leading to the transcription of genes that mitigate oxidative damage, support cellular redox balance, and enhance survival mechanisms (Li B. et al., 2020; Lou et al., 2021). The interplay between NRF2 and the enzyme CHAC1 is crucial in the regulation of cellular redox homeostasis and response to stress. CHAC1 is induced by the integrated stress response (ISR) effector ATF4 under stress conditions such as amino acid starvation or oxidative stress. CHAC1 catalyzes the degradation of GSH into 5-oxoproline and cysteinylglycine. This GSH depletion has a dual effect: it triggers oxidative stress within the cell, thereby stabilizing and activating NRF2. The activation of NRF2 by this mechanism enhances the transcription of genes involved in antioxidant defense and cystine/glutamate exchange, particularly those coding for components of the xCT antiporter (such as SLC7A11), which imports cystine in exchange for glutamate. This exchange is vital for replenishing intracellular cysteine levels, synthesizing new GSH, and maintaining redox balance. Hence, the degradation of GSH by CHAC1 not only induces NRF2 activation but also creates a feedback loop where NRF2 enhances the cell’s capability to cope with oxidative stress by regulating cystine uptake and antioxidant responses. This interplay ensures that cells can adapt to and survive under adverse conditions by fine-tuning their redox environment and metabolic state (Kreß et al., 2023). Nrf2 knockout mice exhibited higher levels of ChaC1 expression during LPS/D-GalN-induced ALF compared to wild-type controls, indicating that the absence of Nrf2 enhances ChaC1 activity. This relationship underscores the protective role of Nrf2 in controlling ChaC1-mediated ferroptosis, as activation of Nrf2 can reduce oxidative stress and inhibit ChaC1, thereby alleviating liver injury and improving survival outcomes in ALF (Huang et al., 2022). Taken together, these data show that NRF2 and ChaC1 negatively affect the expression of the other.

##### 3.1.1.6 p63

p63 is a transcription factor known as a master regulator of epidermal development and keratinocyte (KC) differentiation. It functions either as an activator or repressor of gene transcription and is highly expressed in proliferating basal cells of the epidermis. In the context of atopic dermatitis (AD), p63 is crucial for mediating the repression of KC differentiation induced by type-2 cytokines IL-4 and IL-13. p63 was identified as a direct effector in this process, where its knockdown reverses the inhibition caused by these cytokines. Specifically, p63 regulates the expression of ChaC1, a gene known to negatively regulate Notch signaling, by binding to its promoter region. This regulation by p63 is critical for the cytokine-mediated repression of ChaC1, highlighting its role in altering cell fate decisions and contributing to the impaired differentiation seen in AD (Brauweiler et al., 2021).

##### 3.1.1.7 STAT3

STAT3 negatively regulates the expression of CHAC1 in colorectal cancer cells. Propofol treatment, which downregulates STAT3, leads to the upregulation of CHAC1. This suggests that CHAC1 expression is inhibited by STAT3, and reducing STAT3 levels through propofol treatment releases this inhibition, thereby increasing CHAC1 expression. Elevated CHAC1 is associated with the induction of ferroptosis, highlighting the role of STAT3 as a modulator of CHAC1 and ferroptosis in colorectal cancer cells (Zhao and Chen, 2021).

##### 3.1.1.8 FOXO1

FOXO1 (Forkhead Box O1) is a transcription factor belonging to the forkhead family of proteins, characterized by a distinct forkhead DNA-binding domain. It plays a crucial role in regulating various cellular processes, including metabolism, cell cycle progression, apoptosis, and oxidative stress response. The cooperation between FOXO1, C/EBPδ, and ATF4 significantly affects the expression of ChaC1. FOXO1 activation leads to the upregulation of both C/EBPδ and ATF4, which then coordinate to enhance the transcription of target genes, including ChaC1. This coordinated action amplifies the expression of ChaC1 during muscle atrophy conditions such as fasting. ChaC1 is associated with the regulation of cellular redox states and stress responses, contributing to the proteolytic and catabolic processes that underlie muscle degradation. Therefore, the FOXO1-C/EBPδ-ATF4 axis not only directly drives proteolysis through the ubiquitin-proteasome system but also indirectly supports these processes by elevating stress response genes like ChaC1 (Oyabu et al., 2022; Tabnak et al., 2023).

##### 3.1.1.9 P53

TP53 is a tumor suppressor gene that plays a critical role in preventing cancer formation by regulating cell cycle, DNA repair, and apoptosis. The gene encodes for the p53 protein, which acts as a guardian of the genome by maintaining cellular integrity in response to DNA damage. When gastric epithelial cells are infected by *Helicobacter pylori*, particularly cagA-positive strains, there is an overexpression of CHAC1. This overexpression reduces intracellular GSH levels, causing an imbalance in the cellular redox state and a consequent increase in ROS. These elevated ROS levels damage oxidative DNA, prominently affecting the TP53 tumor suppressor gene. Mutations within TP53, critical in cellular apoptosis and tumor suppression, are thus induced by the oxidative stress mediated by CHAC1 activity. This mechanistic pathway highlights how *H. pylori* infection can contribute to the carcinogenic process in gastric cells by manipulating CHAC1 expression and activity (Wada et al., 2018).

### 3.2 Transmembrane proteins and CHAC1

#### 3.2.1 Notch

**FIGURE 2:**
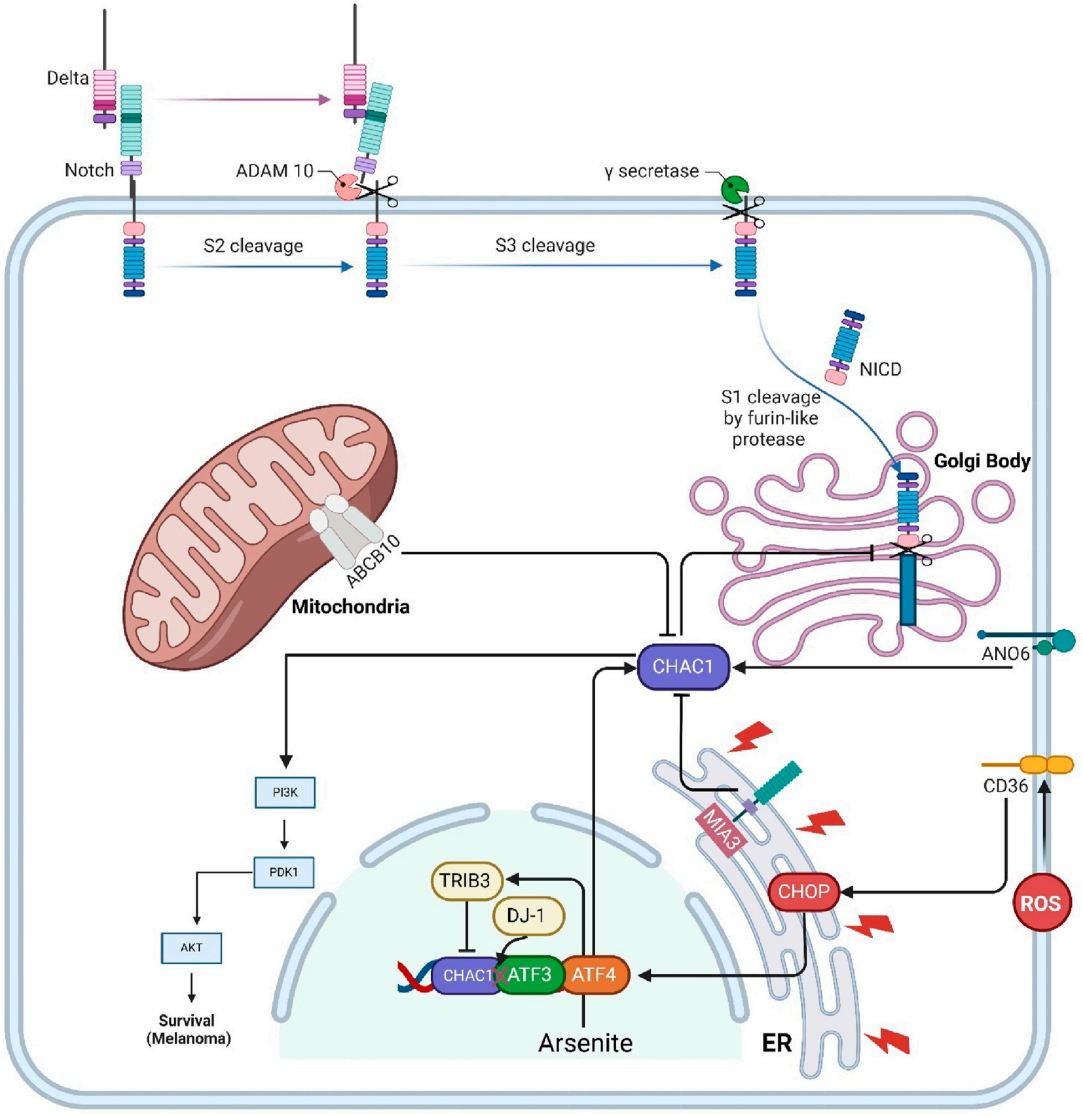
This figure illustrates the interactions and regulatory mechanisms involving CHAC1 and several key transmembrane proteins and protein kinases within cellular pathways. Notch signaling is regulated by CHAC1, which inhibits its maturation, thus reducing cell proliferation and promoting differentiation. MIA3 enhances CHAC1 expression, leading to GSHdegradation and cancer cell survival in HCC. CD36 influences CHAC1 through ER stress responses, while ANO6 induces ferroptosis in GISTs by upregulating CHAC1. Abcb10 affects CHAC1 via nutrient stress responses, and TRIB3 mitigates arsenite toxicity by inhibiting CHAC1 expression. CHAC1 also enhances the PI3K/AKT pathway, promoting cancer cell survival in uveal melanoma. Additionally, DJ-1 regulates CHAC1 to protect against oxidative stress by maintaining GSHlevels. This network underscores CHAC1’s pivotal role in various cellular processes, including interactions with protein kinases, and highlights its potential as a therapeutic target.

Notch is a highly conserved transmembrane protein crucial for regulating cell fate decisions, differentiation, proliferation, and apoptosis during development and in adult tissues. In the canonical Notch pathway, immature Notch receptors on the cell surface interact with ligands such as Delta-like and Jagged. This interaction triggers a series of proteolytic cleavages, beginning with a furin-like protease cleavage (S1) in the Golgi, followed by additional cleavages by ADAM metalloproteases and γ-secretase, releasing the Notch intracellular domain (NICD). NICD then translocates to the nucleus, where it converts the C-promoter binding factor-1 (CBF-1) complex from a transcriptional repressor to an activator, promoting the expression of Notch target genes. CHAC1 regulates this pathway by specifically inhibiting the S1 cleavage of Notch. CHAC1 binds to the S1 cleavage site on the Notch extracellular domain, preventing Notch from maturing and being presented on the cell surface. By maintaining Notch in its immature full-length form, CHAC1 effectively reduces Notch signaling, thereby promoting neuronal differentiation and neurogenesis. This regulation by CHAC1 highlights a critical non-canonical mechanism that modulates Notch activity and neural development (Figure 2) (Chi et al., 2012; Chi et al., 2014). CHAC1 is identified as a critical mediator in the cytotoxic effects of temozolomide (TMZ) on glioma cells, particularly through its interaction with the Notch3 pathway. TMZ treatment results in the upregulation of CHAC1, which subsequently interacts with and inhibits the Notch3 protein. This inhibition is crucial as it reduces the activation of Notch3’s intracellular domain (NICD), leading to decreased Notch3-mediated signaling pathways that are typically involved in glioma cell survival and proliferation. Thus, CHAC1 acts as a suppressor of Notch3, enhancing the apoptotic and cytotoxic effects of TMZ on glioma cells. This interaction between CHAC1 and Notch3 offers potential therapeutic insights, suggesting that targeting CHAC1 could amplify TMZ’s efficacy against glioblastoma by mitigating Notch3 activity (Chen P.-H. et al., 2017).

#### 3.2.2 MIA3

MIA3, also known as TANGO1 (Golgi transporter component protein), is a membrane protein localized at the ER and is implicated in cancer development and progression. In hepatocellular carcinoma (HCC), MIA3 is found to be overexpressed, promoting cancer cell proliferation, migration, and invasion while inhibiting apoptosis. MIA3 exerts its oncogenic effects by interacting with CHAC1. The binding of MIA3 to CHAC1 increases CHAC1 expression, leading to enhanced degradation of GSH, a critical antioxidant that helps maintain cellular redox balance. This degradation of GSH disrupts the cellular redox state, thereby facilitating cancer cell survival and growth. The study confirmed the physical interaction between MIA3 and CHAC1 through coimmunoprecipitation and confocal microscopy, highlighting the MIA3/CHAC1/GSH axis as a key mechanism driving HCC progression and a potential target for therapeutic intervention (Wanbiao et al., 2023).

#### 3.2.3 CD36

CD36, also known as fatty acid translocase (FAT), is a multifunctional membrane glycoprotein expressed on the surface of various cell types, including adipocytes, muscle cells, platelets, macrophages, and endothelial cells. CD36 plays a crucial role in the uptake and metabolism of long-chain fatty acids, contributing to lipid homeostasis. It also functions as a receptor for oxidized low-density lipoprotein (oxLDL), which is implicated in the development of atherosclerosis. Additionally, CD36 is involved in the recognition and phagocytosis of apoptotic cells, contributing to immune responses and inflammation (Rupert and Kolonin, 2022). CD36 regulates CHAC-1 expression through its involvement in the endoplasmic reticulum stress response (ERSR) following spinal cord injury. CD36 activation by lipid peroxidation products, like 4-hydroxy-trans-2-nonenal (4HNE), leads to the induction of ER stress markers such as phospho-ATF4 and CHOP in endothelial cells. This stress response triggers the upregulation of pro-apoptotic proteins, including CHAC-1. In CD36−/− mice, the absence of CD36 results in reduced phosphorylation of eIF2α and lower levels of phospho-ATF4 and CHOP, leading to significantly decreased CHAC-1 expression. Thus, CD36 facilitates the detection of misfolded proteins in the ER and activates the downstream signaling cascade that includes CHAC-1 induction (Myers et al., 2014).

#### 3.2.4 ANO6

ANO6, also known as TMEM16F, is a transmembrane protein involved in various cellular processes, including apoptosis, pyroptosis, and ferroptosis. In gastrointestinal stromal tumors (GISTs), ANO6 is found to be downregulated, and its overexpression inhibits tumor growth. ANO6 exerts its anti-tumor effects by inducing cell death through ferroptosis. Specifically, ANO6 upregulates ChaC1, associated with ferroptosis, by increasing intracellular iron and ROS levels, promoting lipid peroxidation and cell death. This regulatory pathway highlights ANO6 as a potential therapeutic target for treating GISTs by leveraging its role in ferroptosis and ChaC1 regulation (Wang et al., 2024).

#### 3.2.5 Abcb10

Abcb10 is a mitochondrial ATP-binding cassette (ABC) transporter protein primarily involved in heme synthesis and erythroid cell differentiation. It is abundant in tissues with high rates of hematopoiesis, such as the fetal liver and adult bone marrow. Abcb10 plays a crucial role in the cellular export of biliverdin, a breakdown product of heme, which is necessary for maintaining cellular redox homeostasis and effective hemoglobinization during erythroid differentiation. When Abcb10 function is lost, as evidenced by studies using CRISPR/Cas9 gene editing in erythroleukemia cell lines, there are marked disruptions in mitochondrial function and cellular metabolism. One of the downstream effects of Abcb10 loss is the activation of a nutrient stress response mediated by the transcription factor ATF4, which regulates the expression of various genes involved in the cellular stress response. Among these is ChaC1, which is upregulated in the absence of Abcb10. ChaC1 contributes to the degradation of GSH, influencing cellular oxidative stress and response mechanisms. Thus, Abcb10 indirectly regulates ChaC1 by influencing the nutrient stress response pathways activated during mitochondrial and metabolic distress, highlighting its integral role in maintaining cellular homeostasis and stress responses (Miljkovic et al., 2023).

### 3.3 Protein kinases

#### 3.3.1 TRIB3

TRIB3 is a pseudokinase involved in cellular stress responses, particularly in mitigating the toxic effects of arsenite exposure. In mouse embryonic fibroblasts (MEFs), arsenite stress induces TRIB3 expression through the ATF4 pathway. TRIB3 helps cells resist arsenite-induced death by inhibiting the expression of CHAC1, a glutathione-degrading enzyme. TRIB3-deficient cells exhibit elevated CHAC1 levels, leading to increased GSH consumption and higher susceptibility to arsenite toxicity. This effect is mediated through specific regulatory elements in the CHAC1 promoter that are sensitive to both arsenite and ATF4, with TRIB3 suppressing CHAC1 transcription by interfering with ATF4’s transactivation capabilities. Silencing CHAC1 in TRIB3-deficient cells restores GSH levels and reduces arsenite-induced cell death, highlighting the critical role of the TRIB3/CHAC1 axis in regulating cell survival under arsenite stress (Figure 2) (Örd et al., 2016).

#### 3.3.2 AKT

AKT, also known as Protein Kinase B (PKB), is a crucial signaling molecule in the phosphoinositide 3-kinase (PI3K) pathway, which plays a significant role in regulating processes such as cell proliferation, survival, growth, and metabolism. It is activated when a signaling molecule binds to a receptor on the cell surface, leading to PI3K activation and subsequent production of phosphatidylinositol (3,4,5)-trisphosphate (PIP3). PIP3 serves as a docking site for AKT, enabling its activation by phosphorylation. The study concerning CHAC1 and uveal melanoma suggests that CHAC1 influences AKT activity indirectly by affecting this pathway. When CHAC1 expression is silenced, the phosphorylation levels of AKT (and thus its activation state) decrease, indicating that CHAC1 may enhance the PI3K/AKT signaling pathway, promoting cell proliferation and survival in cancerous cells. This regulatory relationship highlights CHAC1’s potential role in modulating oncogenic signaling pathways, making it a candidate for targeted therapy in diseases like uveal melanoma where PI3K/AKT signaling is dysregulated (Liu et al., 2019).

### 3.4 DJ-1

DJ-1 is a protein encoded by the PARK7 gene, which is implicated in autosomal recessive early-onset Parkinson’s disease (PD). It belongs to the ThiJ/PfpI protein family and plays a significant role in protecting cells against oxidative stress. DJ-1 is highly conserved across species and typically exists in the cytoplasm but can translocate to the nucleus and mitochondria under oxidative stress. It functions in various cellular processes, including regulating transcription, antioxidative responses, and maintaining mitochondrial homeostasis. DJ-1 regulates CHAC1, a glutathione-degrading enzyme, by interacting with ATF3. DJ-1 binds to the basic leucine zipper domain of ATF3, inhibiting ATF3’s ability to bind to the CHAC1 promoter. This interaction prevents the transcription of CHAC1, thereby reducing CHAC1 levels and subsequently decreasing the degradation of GSH. By downregulating CHAC1 expression, DJ-1 helps maintain higher intracellular levels of GSH, thus enhancing the cell’s antioxidant capacity and protecting neurons from oxidative damage. This regulatory mechanism underscores DJ-1’s crucial role in cellular defense against oxidative stress, highlighting its potential as a therapeutic target for neurodegenerative diseases and conditions involving oxidative damage (Ge et al., 2022; Wang H. et al., 2022).

### 3.5 SETD1B

SETD1B is a histone methyltransferase identified as a critical mediator of mycolactone-induced cell death. It enhances gene expression by methylating histone H3 at lysine 4 (H3K4), influencing chromatin structure and gene transcription. In mycolactone toxicity, SETD1B regulates the expression of CHAC1. When SETD1B is functional, mycolactone exposure leads to the upregulation of CHAC1, resulting in decreased GSH levels and increased oxidative stress, ultimately causing apoptosis. In SETD1B knockout cells, CHAC1 is not upregulated, preventing GSH depletion and conferring resistance to mycolactone-induced cytotoxicity (Figure 2) (Förster et al., 2020).

### 3.6 Non-coding RNAs

**FIGURE 3:**
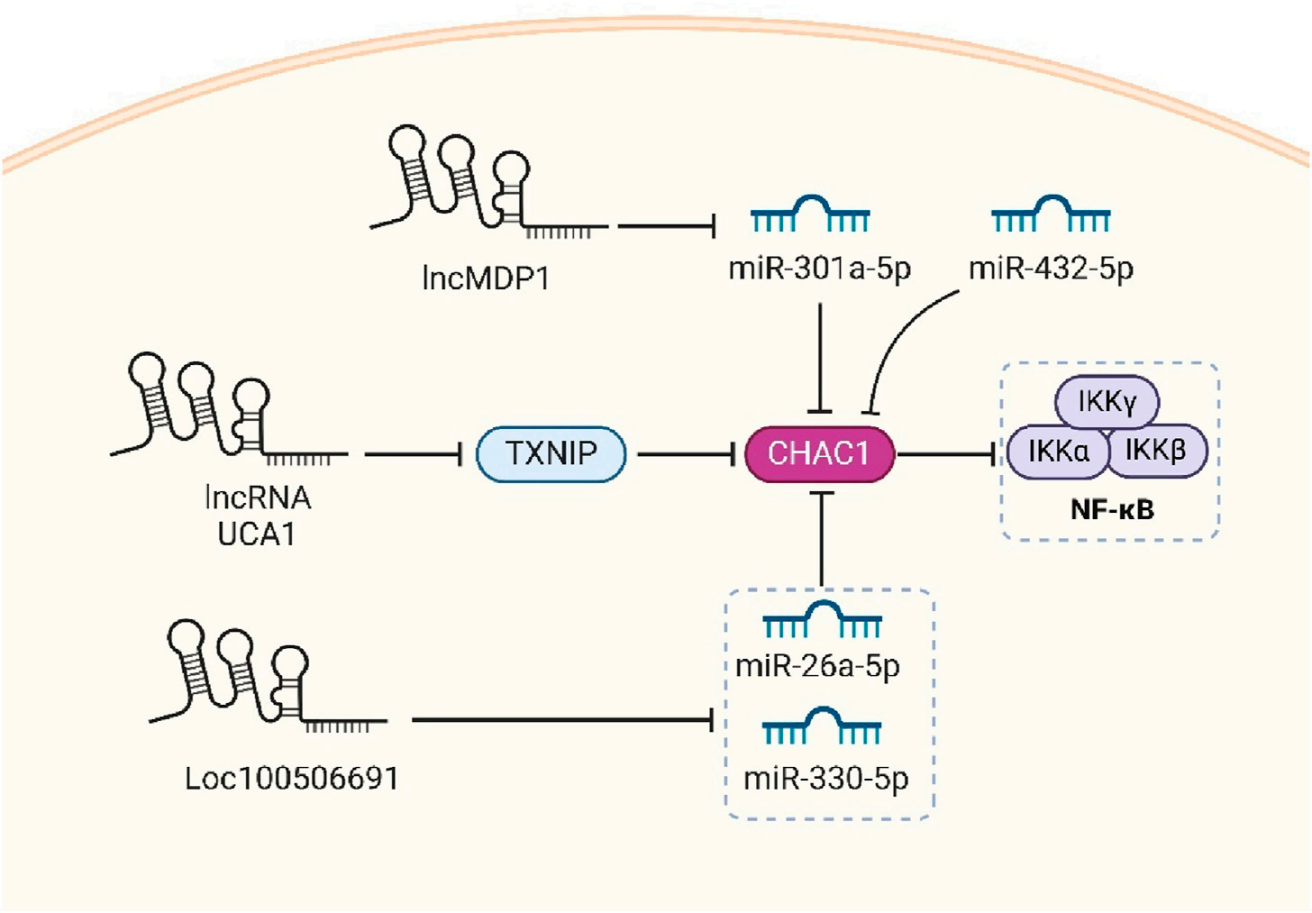
The complex regulatory interactions between lncRNAs and microRNAs in the modulation of CHAC1 expression, which is linked to various cellular processes and diseases. The lncRNAs lncMDP1 and lncRNA UCA1, along with Loc100506691, influence CHAC1 expression indirectly through their interactions with specific miRNAs. For instance, lncMDP1 acts as a sponge for miR-301a-5p, preventing it from inhibiting CHAC1, thereby promoting muscle development. Similarly, UCA1 downregulates TXNIP, which in turn decreases CHAC1 expression, contributing to drug resistance in lung adenocarcinoma. Loc100506691 regulates miR-26a-5p and miR-330-5p, both targeting CHAC1, affecting gastric cancer cell proliferation. Additionally, miR-432-5p and miR-301a-5p directly target CHAC1, impacting processes such as chemoresistance and inflammation. This intricate network highlights potential therapeutic targets for various diseases, including cancer and inflammatory conditions.

Non-coding RNAs (ncRNAs), encompassing long non-coding RNAs (lncRNAs) and microRNAs (miRNAs), play crucial roles in gene regulation without being translated into proteins. LncRNAs, typically exceeding 200 nucleotides, not only regulate gene activity near their synthesis site or at distant locations but also act as “sponges” to bind miRNAs, thereby inhibiting their ability to regulate other target mRNAs. This sponging effect prevents miRNAs from binding to their usual mRNA targets, thus modulating the gene expression landscape. MiRNAs, about 22 nucleotides in length, typically target the 3′untranslated regions (3′UTRs) of mRNAs. This interaction leads to mRNA degradation or repression, forming a sophisticated regulatory network that is vital for proper cellular function and development, and is often implicated in various diseases (Figure 3) (Tabnak et al., 2021b; Tabnak et al., 2021c; Dabbaghi et al., 2023; Yang W. et al., 2023; Zhou et al., 2022).

#### 3.6.1 lncRNA UCA1

Research on the role of lncRNA UCA1 in cisplatin resistance in lung adenocarcinoma outlines several mechanisms through which UCA1 influences the expression of various genes, including ChaC1. Specifically, UCA1 is found to be highly expressed in cisplatin-resistant lung adenocarcinoma cells, and its expression correlates with the downregulation of several mRNAs, including ChaC1. The study reveals that UCA1 overexpression leads to decreased expression of TXNIP, which is significantly associated with the downregulation of ChaC1. This suggests that UCA1 may affect ChaC1 indirectly through its regulation of TXNIP. The downregulation of ChaC1, along with other genes, appears to contribute to the mechanisms by which lung adenocarcinoma cells develop resistance to cisplatin, highlighting the UCA1-TXNIP axis as a potential therapeutic target for overcoming drug resistance (Zhou et al., 2020).

#### 3.6.2 miR-26a-5p

The regulation of CHAC1 by miR-26a-5p plays a crucial role in mitigating inflammatory responses in renal cells, particularly within the context of diabetic kidney disease (DKD). Research indicates that miR-26a-5p directly targets the 3′-untranslated region (UTR) of CHAC1, a gene significantly involved in inflammation-related diseases. By knocking out Rab27a, a key gene in exosome regulation, miR-26a-5p expression in HK-2 cells (a human kidney cell line) rebounds, leading to a decrease in CHAC1 expression. This interaction between miR-26a-5p and CHAC1 effectively reduces the activation of the NF-κB signaling pathway, thus dampening the inflammatory response. The miR-26a-5p/CHAC1/NF-kB axis highlights a potential therapeutic target, suggesting that enhancing miR-26a-5p levels could be beneficial in treating conditions characterized by excessive inflammation, such as DKD (Li S. et al., 2020).

#### 3.6.3 lncMDP1/miR-301a-5p

Researchers investigated the role of lncRNA lncMDP1 in chicken skeletal muscle development. They discovered that lncMDP1 acts as a molecular sponge by adsorbing miR-301a-5p, a microRNA known to bind and regulate the expression of CHAC1, a gene implicated in muscle regeneration and growth. By adsorbing miR-301a-5p, lncMDP1 effectively prevents the miRNA from binding to CHAC1 mRNA, thus alleviating miR-301a-5p′s inhibitory impact on CHAC1 expression. This interaction allows CHAC1 to be expressed at higher levels, which in turn promotes myoblast proliferation and differentiation, crucial processes in muscle development and regeneration. This regulatory mechanism underscores the intricate molecular interactions that control muscle development and offers insights into potential therapeutic strategies for enhancing muscle regeneration (Chen B. et al., 2024).

#### 3.6.4 miR-432-5p

In a study on prostate cancer, cancer-associated fibroblasts (CAFs) were found to secrete exosomal miR-432-5p, which plays a crucial role in promoting chemoresistance in prostate cancer cells by targeting CHAC1. CHAC1 is involved in the regulation of ferroptosis. miR-432-5p binds to the 3′UTR of the CHAC1 gene, suppressing its expression. This suppression leads to the accumulation of GSH, which in turn activates GPX4 and prevents lipid peroxidation, thereby inhibiting ferroptosis. This mechanism allows cancer cells to resist cell death and contributes to the development of resistance to docetaxel, a common chemotherapy drug used in advanced prostate cancer treatment (Zhao et al., 2024).

#### 3.6.5 Loc100506691

Loc100506691 is found to be overexpressed in gastric cancer tissues compared to adjacent normal tissues, and this overexpression correlates with poor patient survival. Metformin, a diabetes medication with anti-tumor properties, is shown to suppress the expression of Loc100506691. This suppression leads to the induction of cell cycle arrest and inhibition of cancer cell proliferation. Loc100506691 negatively regulates CHAC1 by influencing the expression of specific microRNAs (miR-26a-5p and miR-330-5p), which target CHAC1 directly. By controlling these microRNAs, Loc100506691 effectively suppresses CHAC1 expression, thereby promoting cancer cell proliferation and affecting cell motility. Thus, the pathway involving Loc100506691 and CHAC1 presents a potential target for therapeutic intervention in gastric cancer, leveraging metformin’s regulatory impact on this lncRNA (Tseng et al., 2021).

## 4 Cellular mechanisms regulated by ChaC1

**FIGURE 4:**
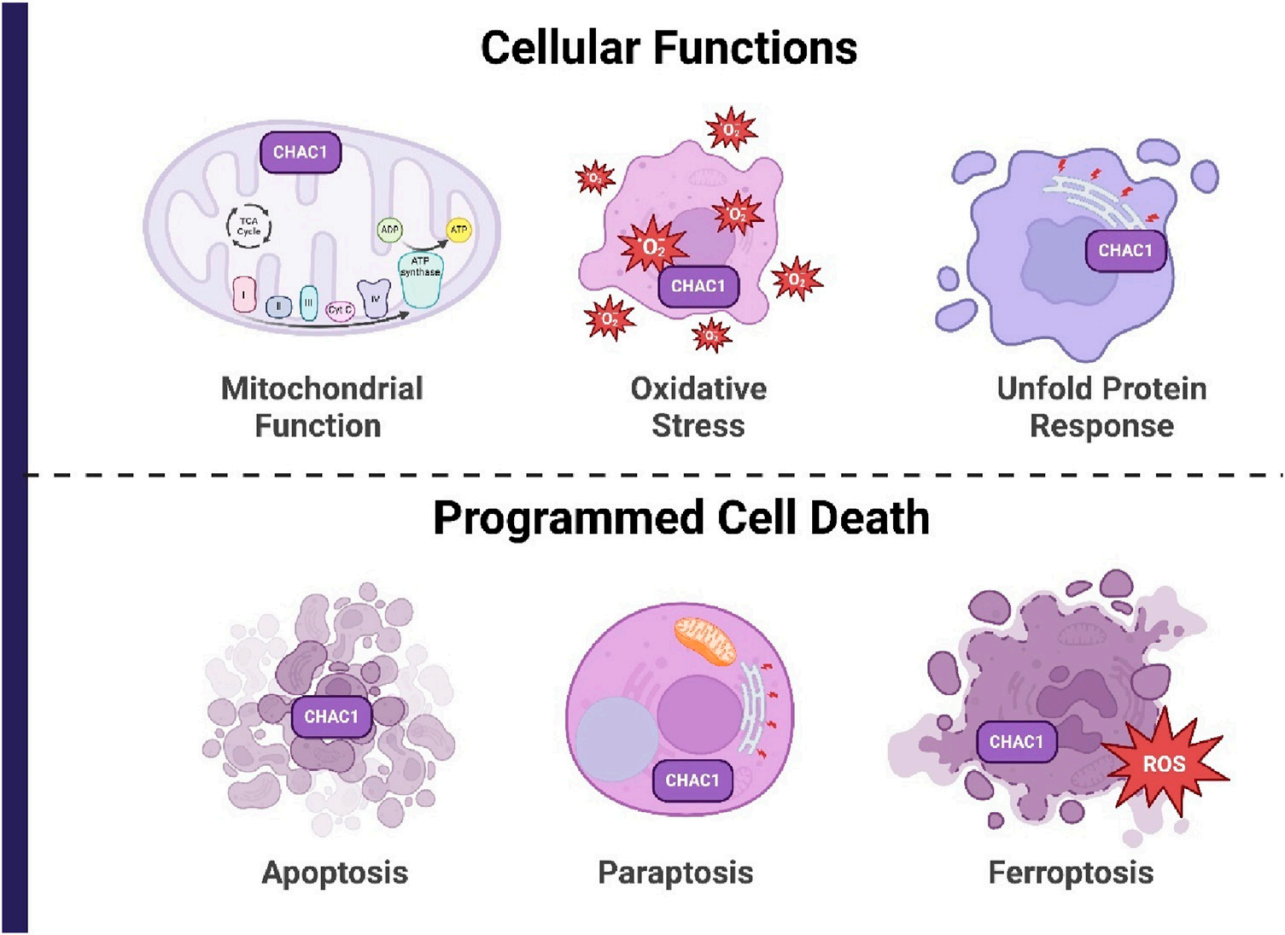
Cellular mechanisms regulated by CHAC1, categorized into cellular functions and programmed cell death. CHAC1 degrades GSH, increasing oxidative stress and disrupting redox balance, which affects mitochondrial function and triggers the UPR. This leads to various forms of programmed cell death, including apoptosis, paraptosis, and ferroptosis, by enhancing oxidative stress and depleting cellular antioxidants.

CHAC1 catalyzes the breakdown of GSH into 5-oxo-proline and Cys-Gly, thereby reducing intracellular GSH levels. This reduction in GSH disrupts the redox balance within cells, leading to increased oxidative stress. CHAC1 is primarily regulated by the UPR pathway, specifically the PERK/eIF2α/ATF4/ATF3/CHOP cascade, which is activated under conditions of ER stress. CHAC1 expression is induced in response to various stresses, including oxidative stress, amino acid deprivation, and viral infection. By depleting GSH, CHAC1 enhances cellular oxidative stress, promoting apoptosis, ferroptosis, and necroptosis. In cancer cells, CHAC1 has been shown to influence the response to chemotherapy by modulating redox homeostasis and contributing to the induction of cell death pathways. Additionally, CHAC1 can interact with other cellular pathways, such as the GCN2/eIF2α/ATF4 pathway, further integrating stress responses with cellular metabolism and apoptosis. In this section, we review the most important cellular mechanisms regulated by ChaC1 (Figure 4) (Zhang et al., 2024).

### 4.1 Oxidative stress

CHAC1 plays a significant role in oxidative stress, particularly under conditions that induce apoptosis, such as heat stress (HS) in cells. Under HS, CHAC1 is notably upregulated as a response to the activation of the ATF4-CHOP signaling pathway. This pathway is critical in the UPR triggered by ER stress, which itself is exacerbated by oxidative stress conditions. CHAC1’s primary function involves the degradation of GSH, a crucial antioxidant. The upregulation of CHAC1 leads to the depletion of GSH levels, weakening the cell’s antioxidant defenses and enhancing oxidative stress. This depletion of GSH is crucial as it diminishes the cell’s ability to neutralize ROS, thereby promoting oxidative damage and apoptosis. Further, the interaction between CHAC1 and the ATF4-CHOP pathway elucidates a feedback loop where oxidative stress enhances ER stress, which in turn aggravates oxidative stress through CHAC1-mediated GSH depletion. This crosstalk underscores the detrimental spiral of increasing stress and cell death, illustrating how CHAC1 not only responds to oxidative stress but also amplifies it, leading to cell apoptosis in conditions like heat stress. This intricate relationship highlights CHAC1 as a pivotal player in the cellular response to oxidative stress, linking metabolic and stress response pathways in a manner that can critically influence cell fate under stress conditions (Cui et al., 2021).

### 4.2 Unfolded protein response

CHAC1 plays a crucial role in the UPR, specifically within the ATF4-ATF3-CHOP signaling pathway. This pathway is activated when cells experience stress related to the accumulation of unfolded proteins in the ER. As a response, CHAC1 is upregulated, primarily influenced by the transcription factor ATF4, and acts downstream of ATF3 and CHOP. These factors collectively mediate cellular responses aimed at restoring normal function by enhancing the protein folding capacity and reducing ER stress. CHAC1 does not respond to other UPR pathways mediated by XBP1 or ATF6, highlighting its specificity in the ATF4-related stress response. The activation of CHAC1 by this pathway leads to increased apoptosis, as evidenced by its role in enhancing apoptosis markers such as TUNEL, PARP cleavage, and AIF nuclear translocation. This selective activation underlines CHAC1’s critical function in maintaining cellular homeostasis and its potential involvement in pathological conditions where ER stress is a significant factor (Mungrue et al., 2009). In contrast, the effect of ER-targeted active form (ChaC1CtoS) on the UPR has been investigated. Surprisingly, active ChaC1CtoS, which efficiently depletes GSHin the ER, showed no significant effect on the UPR. This was determined through dual-channel FACS analysis experiments to measure UPR activity via a CHOP::GFP reporter system in mammalian cells. Even under conditions of GSHdepletion, which one might expect to exacerbate ER stress, there was no measurable increase in UPR activity. This finding was consistent across various experimental conditions, including co-expression with a deregulated allele of the ER oxidase ERO1, designed to increase oxidative stress within the ER. The data robustly suggest that despite its role in reducing the ER’s GSHpool, CHAC1 does not influence the UPR, implying that GSHdepletion alone may not be sufficient to disrupt protein folding homeostasis to a degree that activates the UPR. This challenges the conventional view of the critical role of GSH in managing ER stress and maintaining protein-folding homeostasis (Tsunoda et al., 2014).

### 4.3 Mitochondrial function

CHAC1 plays a critical role in maintaining mitochondrial function and inducing ferroptosis under conditions of cysteine starvation in non-small cell lung cancer (NSCLC) cells. When extracellular cystine (the oxidized form of cysteine) is depleted, CHAC1 is highly induced by the ATF4-mediated stress response. CHAC1 degrades GSH into cysteinylglycine, which is then further cleaved to release cysteine. This catabolism is essential for supplying cysteine to the mitochondria, ensuring the continued synthesis of iron-sulfur (Fe-S) clusters. Fe-S clusters are vital cofactors for various mitochondrial enzymes, including those involved in the electron transport chain (ETC). By supporting Fe-S cluster synthesis, CHAC1 helps maintain mitochondrial respiratory function despite the lack of extracellular cystine. However, this maintenance of mitochondrial activity under cysteine starvation is paradoxically detrimental to NSCLC cell survival, as it promotes ferroptosis. Thus, CHAC1 serves a dual role: it preserves mitochondrial function by ensuring a cysteine supply under nutrient stress, but it also facilitates ferroptosis by maintaining ETC activity, highlighting its potential as a therapeutic target in cancer treatment (Ward et al., 2024).

### 4.4 Apoptosis

CHAC1 is implicated in apoptosis, particularly in the context of ER stress and UPR. Upon activation by conditions inducing ER stress, CHAC1 expression is upregulated primarily through the PERK-eIF2α-ATF3/4 signaling pathway, independent of other UPR branches like IRE1-XBP1. The role of CHAC1 in apoptosis is highlighted by its function as a pro-apoptotic factor that can deplete intracellular GSH levels, reducing the cell’s ability to counteract ROS and oxidative stress, thereby promoting apoptotic cell death. This action of CHAC1 aligns with its regulation by transcription factors ATF4 and ATF3, suggesting a tightly controlled mechanism whereby CHAC1 contributes to the cellular apoptosis process, particularly in stress responses (Joo et al., 2015).

### 4.5 Ferroptosis

**FIGURE 5:**
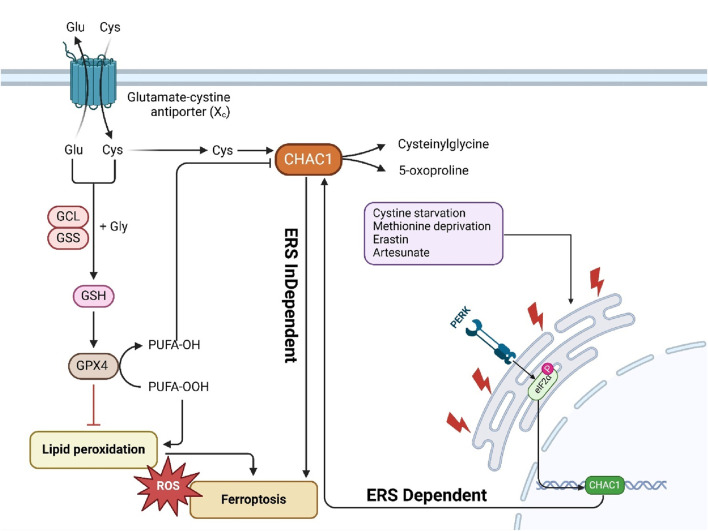
The interplay of CHAC1 with ferroptosis. Central to this process is the glutamate-cystine antiporter system (Xc−), which imports cystine in exchange for glutamate, a critical step for GSH biosynthesis. GSH acts as a reducing substrate for GPX4, which detoxifies lipid hydroperoxides (PUFA-OOH) into non-toxic lipid alcohols (PUFA-OH), thereby preventing lipid peroxidation and ferroptosis. CHAC1 plays a key role in degrading GSH, compromising GPX4 activity, and promoting ferroptosis through increased ROS and lipid peroxidation. This pathway can be activated independently or in response to ER stress, triggered by factors such as cystine starvation, methionine deprivation, erastin, and artesunate. ERS activates the PERK-eIF2α-ATF4 pathway, leading to CHAC1 upregulation and subsequent GSH degradation. Overall, the balance between cystine uptake, GSH synthesis, and GPX4 activity is crucial in determining cell fate concerning ferroptosis, highlighting potential therapeutic targets for manipulating this pathway in diseases like cancer and neurodegenerative disorders.

Ferroptosis is a regulated form of cell death characterized by the accumulation of lipid peroxides, particularly in membrane phospholipids, which occurs when the cellular antioxidant defense is insufficient. This process is critically dependent on the availability of iron and the failure of the selenoenzyme GPX4 to reduce lipid hydroperoxides (LOOH) to non-toxic lipid alcohols (LOH). GSH plays a crucial role in this antioxidant defense as it is the reducing substrate for GPX4. When GSH levels are depleted, GPX4 activity is compromised, leading to unchecked lipid peroxidation and ferroptosis induction. Thus, GSH is essential in maintaining cellular redox balance and preventing ferroptosis by supporting GPX4 in detoxifying lipid peroxides (Ursini and Maiorino, 2020). SLC7A11, also known as xCT, is a critical component of the cystine/glutamate antiporter system Xc^−^, which imports extracellular cystine in exchange for intracellular glutamate. This transport is essential for cancer cells to maintain their antioxidant defense by facilitating cystine uptake, which is subsequently reduced to cysteine and used for GSH biosynthesis. GSH, in turn, plays a pivotal role in detoxifying lipid peroxides via GPX4, thereby preventing ferroptosis, a form of regulated cell death characterized by excessive lipid peroxidation. Overexpression of SLC7A11 in cancer cells suppresses ferroptosis, promoting cell growth and survival by enhancing GSH production and neutralizing oxidative stress. Additionally, SLC7A11-mediated cystine uptake induces metabolic vulnerabilities, including glucose and glutamine dependencies, creating potential therapeutic targets for inducing ferroptosis in cells by disrupting their redox balance and metabolic homeostasis (Koppula et al., 2021). NRF2 plays a crucial role in regulating ferroptosis. It modulates the expression of various genes involved in iron, lipid, and amino acid metabolism, thus maintaining cell redox homeostasis. By activating AREs, NRF2 enhances the transcription of genes that encode for antioxidant proteins and enzymes, such as GPX4, which reduces lipid peroxides and mitigates oxidative stress. Additionally, NRF2 influences iron storage and export by regulating ferritin and ferroportin, and it affects GSH synthesis and recycling through the cystine/glutamate antiporter system and related pathways. Therefore, targeting NRF2-related signaling pathways offers potential therapeutic strategies for inducing or inhibiting ferroptosis in various diseases, including cancer and neurodegenerative disorders (Yan et al., 2023). CHAC1 enhances cystine-starvation-induced necroptosis and ferroptosis in human triple-negative breast cancer (TNBC) cells by degrading GSH. Under cystine starvation, the GCN2-eIF2α-ATF4 pathway is activated, leading to an upregulation of CHAC1 expression. CHAC1 then degrades GSH, a critical antioxidant, resulting in elevated levels of ROS. This increase in ROS triggers mitochondrial dysfunction, further exacerbating oxidative stress and promoting cell death through both necroptosis and ferroptosis pathways (Figure 5) (Chen M.-S. et al., 2017). In Table 1, we present a comparative analysis of other proteins involved in cancer treatment, particularly those influencing ER stress or ferroptosis. By comparing CHAC1 with proteins that share similar functions, this analysis provides context and emphasizes the distinct characteristics of CHAC1.

**TABLE 1:** Comparative analysis of some proteins involved in cancer treatment, particularly those influencing ER stress and ferroptosis.

Protein	Primary function	Role in ferroptosis	Role in ER stress	Therapeutic potential	Key differences	Ref.
CHAC1	Degrades GSH, reducing cellular antioxidant capacity	Promotes ferroptosis by depleting intracellular GSHlevels	Activated during ER stress via the ATF4 pathway, enhancing apoptosis	Potential target to induce ferroptosis in cancer cells by lowering GSHlevels and sensitizing them to oxidative stress	Directly depletes GSH, unlike other proteins that regulate its synthesis or use, contributing to unique control over ferroptosis and redox balance	Zhang et al. (2024)
GPX4	Reduces lipid peroxides, preventing ferroptosis	Directly detoxifies lipid peroxides; its inhibition leads to ferroptosis	Not directly involved in ER stress	Target for ferroptosis induction in cancer cells; inhibition triggers ferroptotic cell death	GPX4 detoxifies lipid peroxides, while CHAC1 lowers GSHlevels, indirectly enhancing ferroptosis by limiting antioxidant defenses	Yang et al. (2014)
SLC7A11	Cystine/glutamate transporter, crucial for GSHsynthesis	Prevents ferroptosis by facilitating cystine uptake for GSHsynthesis	Limited role in ER stress	Target in cancer therapy to induce ferroptosis by blocking cystine uptake	SLC7A11 controls cystine import, while CHAC1 depletes existing GSH, representing different control points in the ferroptosis pathway	Koppula et al. (2021)
ATF4	Regulates amino acid metabolism and cellular stress response	Indirect role in ferroptosis by upregulating CHAC1 and other stress response genes	Central player in the UPR, activating ER stress pathways	Target for modulating cellular stress responses in cancer	ATF4 regulates CHAC1 expression, functioning upstream in the ER stress response, whereas CHAC1 directly acts by degrading GSH	Neill and Masson (2023)
NRF2	Master regulator of antioxidant defense	Protects cells from ferroptosis by upregulating antioxidant genes	Limited involvement in ER stress, primarily oxidative stress	Target to enhance antioxidant responses in cancer therapy	NRF2 boosts antioxidant defenses by increasing GSHsynthesis and usage, contrasting with CHAC1’s role in promoting oxidative stress via GSHdepletion	He et al. (2020)
CHOP (C/EBP Homologous Protein)	Promotes apoptosis during prolonged ER stress	Indirectly involved in ferroptosis by regulating stress response pathways	Key regulator of ER stress-induced apoptosis	Target for inducing cell death in cancers with high ER stress	CHOP primarily triggers apoptosis in response to ER stress, while CHAC1 induces ferroptosis by degrading GSH, linking both to cell death through different mechanisms	Di and Ho (2020)

#### 4.5.1 Ferroptosis inducing agents and CHAC1

A study has investigated the impact of ferroptosis-inducing agents (FIAs) on CHAC1 mRNA expression in human islets. Specifically, treatment with erastin, a known FIA, significantly increased the expression of CHAC1 mRNA compared to non-treated control islets. This indicates that erastin-induced ferroptosis is associated with upregulation of CHAC1. However, another FIA, RSL3, did not significantly affect CHAC1 expression. These findings suggest that the pathway of ferroptosis induction by erastin involves CHAC1-mediated GSHdepletion, while RSL3’s mechanism may differ or bypass this specific pathway (Bruni et al., 2018). In artesunate-induced ferroptosis in Burkitt’s lymphoma (BL) cells, CHAC1 is upregulated as part of the ATF4-CHOP-CHAC1 pathway activation. This upregulation leads to a significant decrease in GSH levels, weakening the cell’s antioxidant defenses and making it more susceptible to lipid peroxidation and oxidative stress. Silencing CHAC1 expression in BL cells has been shown to increase cell viability and resistance to ferroptosis, demonstrating that CHAC1-mediated GSH degradation is a pivotal mechanism through which artesunate exerts its cytotoxic effects (Wang et al., 2019).

#### 4.5.2 ER stress-induced CHAC1 expression and ferroptosis

ER stress-induced CHAC1 expression contributes to ferroptosis by disrupting GSH homeostasis, which is crucial for cellular antioxidant defense. Under ER stress, the integrated stress response (ISR) pathway is activated, leading to the phosphorylation of eIF2α and subsequent upregulation of ATF4, a transcription factor that increases CHAC1 expression. CHAC1 degrades GSH, reducing the GSH/GSSG ratio and impairing the activity of GPX4, an enzyme essential for detoxifying lipid peroxides. The diminished GPX4 activity results in the accumulation of lipid peroxides within mitochondria, triggering ferroptosis. Thus, ER stress-induced CHAC1 expression depletes GSH, compromises GPX4 function, and promotes ferroptotic cell death (Kitakata et al., 2021). While CHAC1 has been primarily associated with ER stress pathways, it can also induce ferroptosis through mechanisms that are independent of ER stress. CHAC1 can induce ferroptosis independently of ER stress, primarily by depleting GSH. This depletion reduces the cell’s ability to neutralize ROS, leading to an imbalance in the redox state and promoting the accumulation of lipid ROS. CHAC1 also disrupts iron homeostasis, increasing free iron levels that catalyze ROS formation through the Fenton reaction. Additionally, the GSH depletion impairs GPX4 activity, as GSH is a necessary cofactor, leading to unchecked lipid peroxidation. Furthermore, CHAC1 can influence the expression of pro-ferroptotic genes, enhancing lipid metabolism and iron uptake/storage, which collectively drive ferroptosis by promoting lipid peroxidation and reducing cellular antioxidant defenses. Through these mechanisms, CHAC1 facilitates ferroptosis via pathways focused on oxidative stress and lipid peroxidation rather than ER stress (Gagliardi et al., 2019).

#### 4.5.3 Possible role of methionine

Prolonged methionine deprivation blocks CHAC1 protein synthesis by interfering with its translation, thereby inhibiting ferroptosis. This process begins with cystine deprivation, which typically induces ferroptosis by depleting intracellular GSH, a critical antioxidant. CHAC1, an enzyme that degrades GSH, is upregulated during cystine deprivation through the activation of the GCN2-eIF2α-ATF4 pathway, enhancing ferroptosis onset. However, when methionine deprivation is prolonged, it disrupts protein synthesis, including that of CHAC1, at the translational level. Although CHAC1 mRNA levels remain elevated, the lack of methionine, essential for initiating protein synthesis, prevents the translation of CHAC1 mRNA into functional protein. Consequently, this disruption in CHAC1 synthesis hinders the accelerated degradation of GSH, maintaining its levels above the threshold needed to prevent ferroptosis. Thus, prolonged methionine deprivation mitigates the cellular stress and oxidative damage that would otherwise lead to ferroptosis, providing a cellular protective mechanism against the induction of this form of cell death (Xue et al., 2023).

#### 4.5.4 Lipid metabolism and CHAC1

Lipids inhibit the degradation of GSH by negatively regulating CHAC1, a key enzyme involved in GSH catabolism and associated with the ER stress response. Specifically, treatment with a lipid mixture (LM), cholesterol (CHOL), and oleic acid (OA) blocked the increase in CHAC1 expression induced by the ferroptosis inducer RSL3 in colorectal cancer cells. This suppression of CHAC1 by lipids prevented the reduction of GSH and maintained the GSH/GSSG ratio, thereby inhibiting ferroptosis. Overexpression of CHAC1 reversed the protective effect of lipids against ferroptosis, confirming that lipids ameliorate ferroptosis through the inhibition of CHAC1-mediated GSH degradation (Zhang et al., 2021).

### 4.6 Paraptosis

Paraptosis is a unique form of programmed cell death distinct from apoptosis, characterized by extensive cytoplasmic vacuolation and the swelling of the ER and mitochondria. Unlike apoptosis, it does not involve nuclear fragmentation, chromatin condensation, or caspase activation. Paraptosis can be induced by various triggers, such as activating the insulin-like growth factor 1 receptor (IGF1R), proteasomal inhibition leading to ER stress, ROS generation, and calcium influx into mitochondria. This alternative cell death pathway offers potential in cancer therapy, especially in cases where cancer cells have developed resistance to apoptosis, by providing a new avenue to target and eliminate cancer cells through distinct biochemical and morphological mechanisms (Hanson et al., 2023). CHAC1 plays a key element in the induction of paraptosis through its involvement in GSH degradation. In the context of dual inhibition of thioredoxin reductase (TrxR1) and the proteasome, CHAC1 is upregulated via the ATF4/CHAC1 axis. This upregulation leads to the degradation of GSH, causing a depletion of intracellular GSH levels. The resulting GSH depletion exacerbates proteotoxic stress by allowing the accumulation of misfolded thiol-containing proteins within the ER and mitochondria. This proteotoxic stress, driven by the imbalance in thiol homeostasis, is a key mechanism through which CHAC1 mediates the paraptotic cell death pathway in breast cancer cells treated with auranofin and proteasome inhibitors (Seo et al., 2023).

## 5 CHAC1 involvement in non-malignant diseases

Oxidative stress, resulting from the excessive accumulation of ROS and reactive nitrogen species (RNS), plays a critical role in the pathogenesis of various non-malignant diseases. It contributes to the onset and progression of metabolic disorders such as diabetes and obesity by damaging cellular structures through lipid peroxidation, protein modification, and DNA damage. In diabetes, oxidative stress exacerbates complications like diabetic retinopathy and nephropathy by impairing cellular functions and promoting inflammatory responses. Additionally, oxidative stress is implicated in cardiovascular diseases, where it induces endothelial dysfunction, promotes atherosclerosis, and impairs cardiac function. Neurodegenerative diseases, including Alzheimer’s and Parkinson’s, are also heavily influenced by oxidative stress, which accelerates the formation of harmful protein aggregates and neuroinflammation (Seo et al., 2023).

**FIGURE 6:**
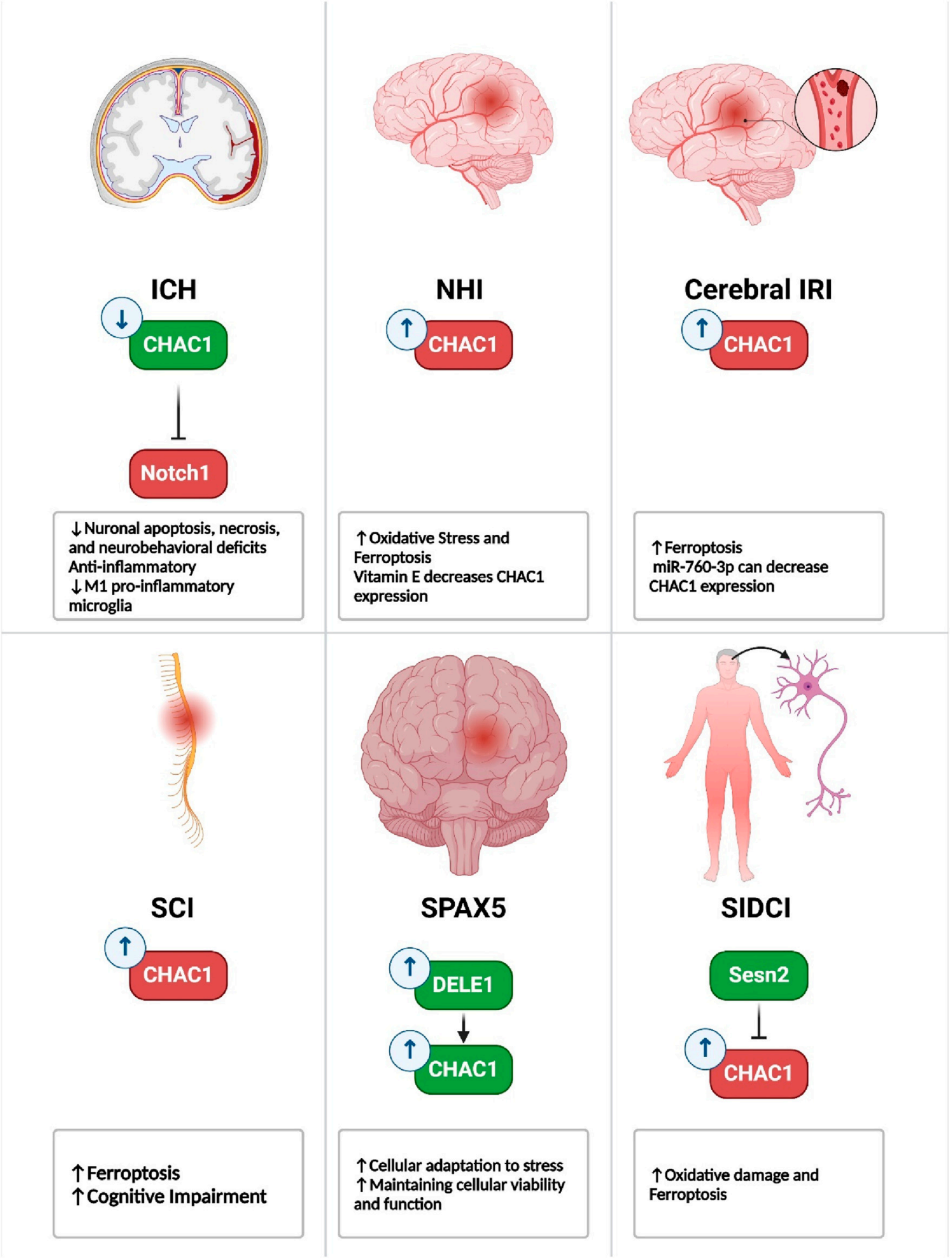
Role of CHAC1 in neurological diseases. Generally, CHAC1 overexpression is associated with disease progression, except in conditions such as ICH and SPAX5.

CHAC1 is a key regulator of cellular stress responses, particularly oxidative stress, apoptosis, and ferroptosis, and it plays a central role in the pathogenesis of a wide range of non-malignant diseases. In neurological conditions like cerebral hemorrhage, neonatal hypoxia-ischemia, and spinal cord injury, CHAC1 exerts both protective and damaging effects. In cerebral hemorrhage, CHAC1 helps mitigate inflammation and neuronal damage by inhibiting the Notch1 signaling pathway, reducing cell death and secondary brain injury. However, in conditions like ischemic stroke and spinal cord injury, CHAC1 promotes neuronal damage by inducing ferroptosis through the depletion of GSH, a critical antioxidant, exacerbating oxidative stress and worsening the injury (Mei et al., 2017; Chen M. et al., 2021; Kolnik et al., 2022; Wang et al., 2023; Kang et al., 2022; Li J. et al., 2021) (Figure 6).

**FIGURE 7:**
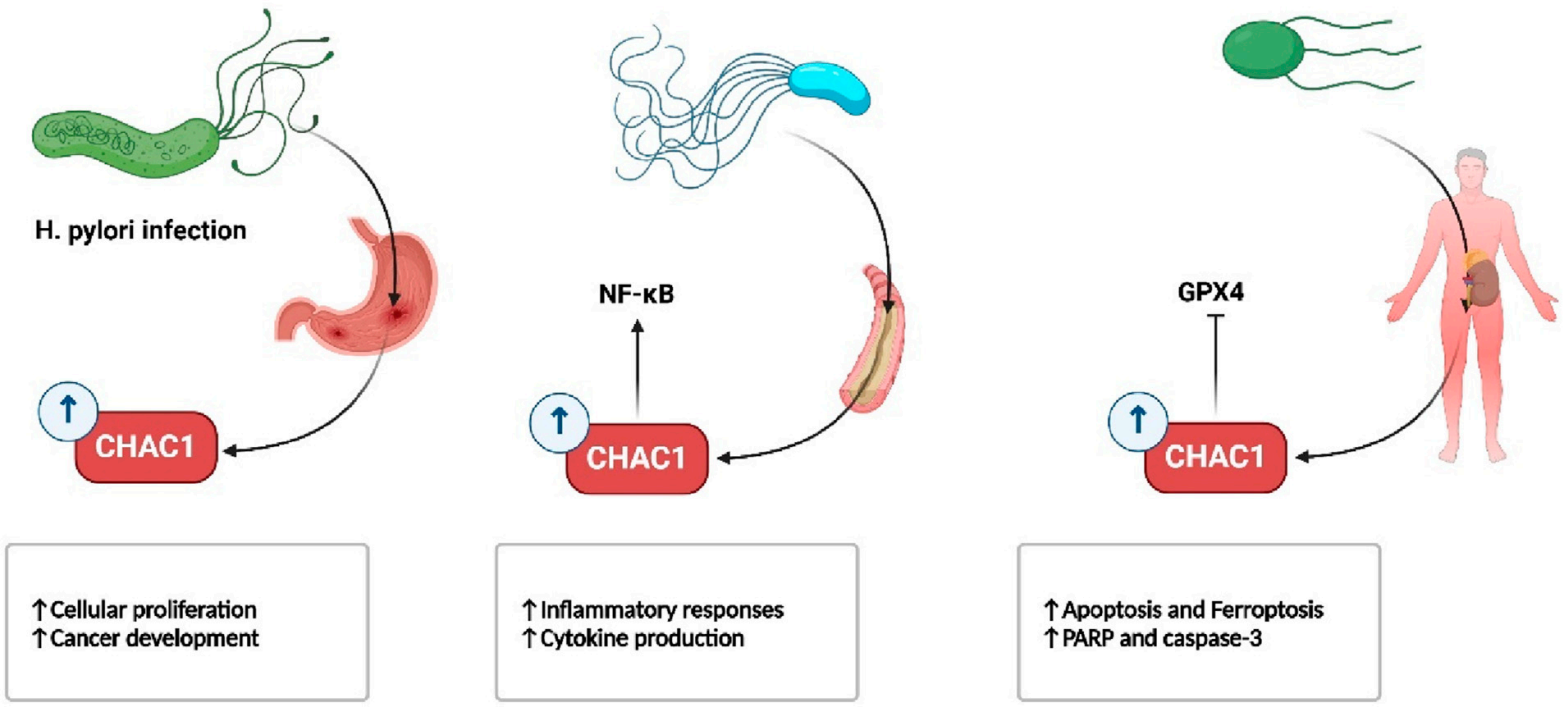
CHAC1 expression is upregulated upon infectious diseases.

In infectious diseases, CHAC1 also plays a role in immune cell dysfunction and apoptosis. During sepsis, for instance, CHAC1 drives ferroptosis in dendritic cells and kidney cells, leading to increased organ damage and worsening outcomes. Its overexpression has been linked to higher susceptibility to oxidative damage in diseases like *Helicobacter pylori* infection and cystic fibrosis. In *H. pylori* infection, CHAC1 contributes to gastric cell oxidative stress and mutations that could potentially lead to cancer, while in cystic fibrosis, reduced CHAC1 levels contribute to chronic inflammation by impairing the body’s ability to regulate oxidative stress (Wada et al., 2018; Ogawa et al., 2019; Perra et al., 2018) (Figure 7).

Metabolic disorders such as diabetes are also affected by CHAC1’s role in β-cell survival. CHAC1 is involved in the stress-induced apoptosis of β-cells, contributing to the progression of both type 1 and type 2 diabetes by impairing insulin production (Juliana et al., 2018).

In muscle-wasting conditions like cancer cachexia, CHAC1 exacerbates oxidative stress by degrading GSH, thereby accelerating muscle degradation and protein breakdown (Li et al., 2023). This highlights its involvement in not just neurological and infectious diseases, but also systemic metabolic conditions.

**FIGURE 8:**
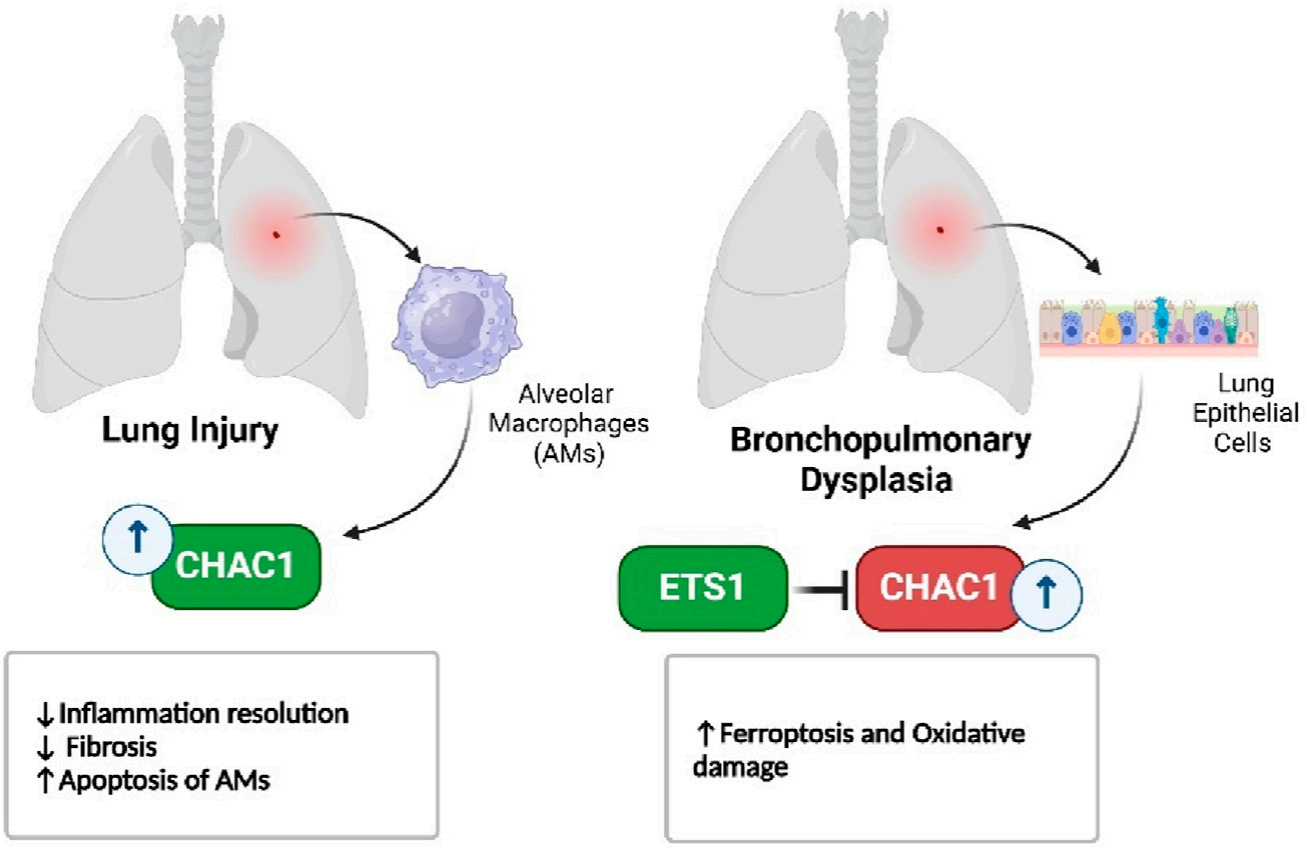
CHAC1’s dual role in lung disorders.

**FIGURE 9:**
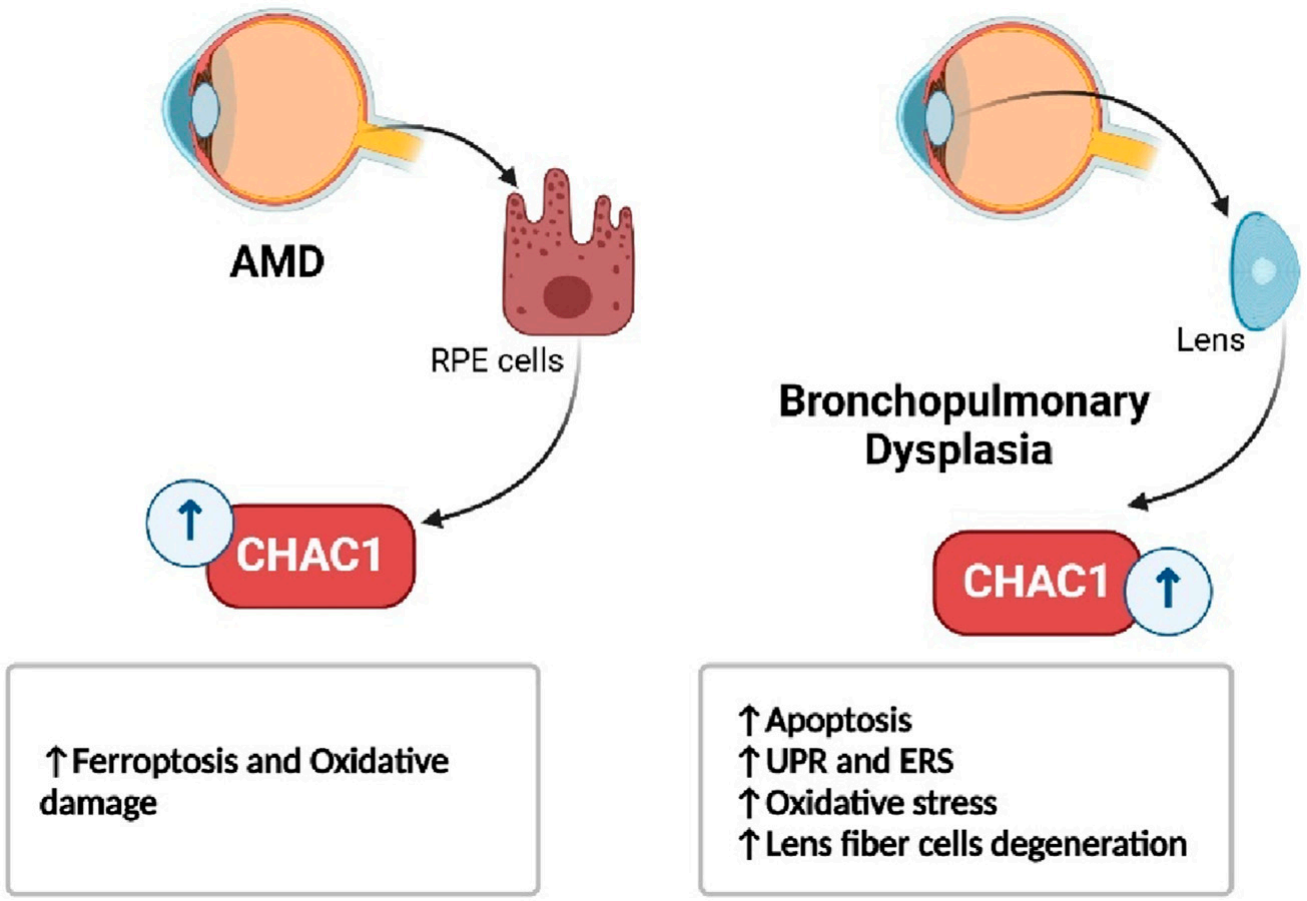
CHAC1’s role in eye disorders.

In respiratory diseases, such as bleomycin-induced lung injury and bronchopulmonary dysplasia, CHAC1 contributes to the resolution of inflammation by promoting the apoptosis of immune cells, but its role in oxidative stress also highlights its damaging potential (Figure 8) (Yang M. et al., 2023; Allawzi et al., 2019). Similarly, in eye disorders like age-related macular degeneration and cataracts, CHAC1 promotes cell death through oxidative stress pathways, making it a significant factor in the progression of these conditions (Figure 9) (Liu et al., 2023a; Zhou et al., 2016).

CHAC1’s role in acute liver injury and atherosclerosis further underscores its involvement in inflammatory and apoptotic pathways. In liver injury, CHAC1’s promotion of GSHdegradation accelerates oxidative stress and liver cell death, while in atherosclerosis, it contributes to plaque instability by promoting apoptosis in lesional macrophages and smooth muscle cells (Xu et al., 2023; Dorweiler et al., 2014). Moreover, in conditions such as endometriosis, CHAC1’s regulation of ferroptosis plays a critical role in controlling the survival of ectopic endometrial cells, with potential implications for treatment (Zou et al., 2024).

In summary, CHAC1 is a crucial mediator of oxidative stress, inflammation, and cell death across various diseases, from neurological and metabolic disorders to infectious, respiratory, and chronic inflammatory conditions. While its activity is often associated with disease progression through the promotion of ferroptosis and oxidative damage, it also holds potential as a therapeutic target, with selective inhibition of CHAC1 offering possible benefits in conditions where its overexpression drives pathology.

In Table 2, we have summarized the role of CHAC1 in the pathogenesis of various non-malignant diseases.

**TABLE 2:** The role of CHAC1 in the pathogenesis of various non-malignant diseases.

Type of disease	Cell lines	Model (in vivo and in vitro)	Effect on ferroptosis	Molecular targets	Highlights (with focus on role of CHAC1)	Ref.
Neurological Diseases						
Intracerebral Hemorrhage (ICH)-induced Secondary Brain Injury (SBI)	Cultured primary rat cortical neurons	Sprague-Dawley (SD) rat model, ICH induced by autologous arterial blood injection	Not directly assessed; focused on apoptosis and inflammation	Notch1 signaling pathway, ChaC1 (Blocks Notch maturation), Notch1-TMIC, NICD	- ChaC1 overexpression inhibited Notch1-TMIC and NICD maturation, reducing apoptosis and inflammation - Knockdown of ChaC1 worsened SBI, increasing apoptosis, necrosis, and inflammatory cytokines (IL-6, IL-1β, TNF-α) - ChaC1’s neuroprotective function depends on the Glu115 residue	Mei et al. (2017)
Intracerebral Hemorrhage (ICH)	Not applicable (*in vivo* study on rats)	Sprague-Dawley (SD) rat model, ICH induced by type IV collagenase	Not directly assessed; focused on apoptosis, inflammation, and microglial activity	AR, JMJD3, Botch, Notch1, NICD	- AR overexpression enhances microglial activation and secondary brain injury - AR represses JMJD3, leading to Botch downregulation and Notch1 activation - JMJD3 suppresses Botch methylation, promoting Notch1 signaling inhibition, reducing microglial activation and brain injury - AR/JMJD3/Botch axis modulates neuroinflammation and apoptosis after ICH.	Chen M. et al. (2021)
Neonatal Hypoxia-Ischemia	Ferret brain slice cultures	Organotypic whole-hemisphere (OWH) ferret brain slice model subjected to oxygen-glucose deprivation (OGD)	Vitamin E decreases oxidative stress, reduces cytotoxicity, and lowers cell death in brain slices, suggesting potential to mitigate ferroptosis	GSH(GSH), CHAC1, SLC7A11, HMOX1, TNF-α, IL-8	Vitamin E preserves GSH levels, decreases markers of oxidative stress (CHAC1, SLC7A11), and inflammation (TNF-α, IL-8), more effective than ferrostatin	Kolnik et al. (2022)
Cerebral Ischemia/Reperfusion (I/R) Injury	Mouse neuroblastoma cell line (N2a), C57/BL6 mice	Mouse middle cerebral artery occlusion (MCAO) model subjected to ischemia/reperfusion injury	ADSC-derived exosomes containing miR-760-3p inhibit ferroptosis in neurons by targeting CHAC1, reducing oxidative stress and lipid peroxidation	CHAC1, miR-760-3p, ACSL4, GPX4, MDA, NeuN	Intranasal administration of exosomes efficiently delivers miR-760-3p to neurons, reduces ferroptosis by downregulating CHAC1, and improves neurological function in ischemic stroke mice	Wang et al. (2023)
Spinal Cord Injury (SCI)	Primary rat spinal cord cells	*In vivo* (Sprague-Dawley rats) *In vitro* (cultured spinal cord cells)	Promotes ferroptosis during secondary SCI	CHAC1, ATF3, XBP1, HMOX1, DDIT3	CHAC1 is upregulated in response to oxidative stress in SCI, facilitating ferroptosis by depleting GSHlevels. CHAC1 is a key player in the GSHdegradation pathway	Kang et al. (2022)
Sepsis Neuronal Injury	Dendritic Cells (DCs)	LPS-induced and CLP	Suppresses ferroptosis via Sesn2 by reducing GSH depletion	ATF4-CHOP-CHAC1 signaling pathway, GPX4, ACSL4, TFRC, xCT	Sesn2 protects DCs from ferroptosis by downregulating the ATF4-CHOP-CHAC1 pathway. CHAC1 degrades GSH, exacerbating ferroptosis. Sesn2 knockout leads to upregulated CHAC1 expression, worsening cell death	Li D. et al. (2021)
	Splenocytes	Sesn2^−/−^ and WT mice	Sesn2 knockout exacerbates ferroptosis	ATF4, CHOP, CHAC1, GPX4, xCT	CHAC1 activation is linked to increased ferroptosis via GSH degradation. Sesn2 inhibits CHAC1, reducing oxidative stress in septic DCs	
Infectious Diseases						
Gastric cancer (*H. pylori* infection)	AGS cells	*In vitro*	Overexpression of CHAC1 leads to GSH depletion, increasing ROS and potentially ferroptosis	CHAC1, TP53, ROS, GSH	*H. pylori* infection (specifically cagA-positive) triggers CHAC1 overexpression, which degrades GSH and accumulates ROS. This increases oxidative stress, leading to somatic mutations in TP53, a key tumor suppressor gene. CHAC1 knockdown via siRNA prevents these mutations, indicating its pivotal role in DNA damage and cancer progression	Wada et al. (2018)
Gastric cancer (due to *H. pylori* infection)	AGS cells (human gastric epithelial cells) and human gastric mucosa tissues	*In vitro*	CHAC1 overexpression leads to GSH depletion, increasing ROS, possibly triggering ferroptosis	CHAC1, TP53, ROS, GSH	*H. pylori* infection induces CHAC1 overexpression, specifically in parietal cells. This degrades GSH(GSH), leading to ROS accumulation, oxidative DNA damage, and somatic mutations in the tumor suppressor gene TP53. CHAC1 overexpression in parietal cells contributes to gastric carcinogenesis	Ogawa et al. (2019)
*Pseudomonas aeruginosa* Induced Cystic Fibrosis (CF)	hAECBs from CF and non-CF patients, NCI-H292, BEAS2-B, A549	*In vitro* (cell cultures of human airway epithelial cells)	Not directly studied in ferroptosis but involves GSHregulation	CHAC1, ATF4, NF-κB, PERK, IL-8, IL-6, CCL2	CHAC1 expression is differentially regulated in CF and non-CF cells. It plays a role in modulating the inflammatory response to *Pseudomonas aeruginosa* (Pa) and its virulence factors. CHAC1 was overexpressed in non-CF cells during infection, indicating its role in managing excessive inflammation in CF, potentially by regulating the NF-κB pathway and inflammatory cytokines (IL-8, IL-6, CCL2). CHAC1 expression through the ATF4 pathway helps prevent overactive inflammation, but its reduction in CF cells may contribute to heightened inflammatory responses. CHAC1 did not induce apoptosis in Pa-infected cells	Perra et al. (2018)
Lung Diseases						
Acute Lung Injury, Pulmonary Fibrosis	Bone marrow–derived macrophages (BMDMs), RAW264.7 macrophages, Recruited Alveolar Macrophages (AM)	*In vivo* (Bleomycin-treated mice, R213G variant and WT), *In vitro* (macrophage cultures)	Not directly studied in ferroptosis but involves GSHregulation via CHAC1	EC-SOD (SOD3), CHAC1, PARP, GSH, OxPAPC, H2O2	CHAC1 is upregulated in recruited alveolar macrophages (AMs) from R213G mice post-bleomycin treatment. CHAC1 promotes apoptosis in macrophages through GSHdegradation, resolving inflammation more rapidly in R213G mice. This effect is associated with increased levels of oxidized phospholipids (OxPAPC) and reactive oxygen species (H2O2) in the alveolar environment. CHAC1 overexpression induces apoptosis via GSHdegradation, which can be attenuated by exogenous GSH. CHAC1-mediated macrophage apoptosis is critical for reducing inflammation and preventing fibrosis in the R213G variant model	Allawzi et al. (2019)
Bronchopulmonary Dysplasia (BPD)	A549 (human alveolar epithelial cells)	*In vivo* (C57BL/6J neonatal mice), *In vitro* (A549 cells)	Suppresses ferroptosis in hyperoxia-induced BPD	ETS1, Nrf2, HO-1, CHAC1, PTGS2, GPX4	CHAC1 expression was upregulated in hyperoxia-induced ferroptosis, contributing to increased ROS and Fe^2^⁺ production. ETS1 overexpression reduced CHAC1 expression, ameliorating ferroptosis	Yang W. et al. (2023)
Eye Related Disorders						
Age-related macular degeneration (AMD)	ARPE-19 (human retinal pigment epithelial cells)	*In vivo* (Laser-induced CNV mice), *In vitro* (ARPE-19 cells)	Promotes ferroptosis in oxidative-damaged RPE cells	CHAC1, GSH, Fe^2^⁺, 4-HNE, ALDH2	CHAC1 upregulated under oxidative stress, depleting GSH and increasing lipid peroxidation and iron accumulation. CHAC1 silencing reversed these effects, showing its pivotal role in ferroptosis	Liu et al. (2023a)
Cataract	None (Mouse model study)	*In vivo* (Mip-mutant mouse model)	Promotes cell death via GSH depletion and oxidative stress	CHAC1, DDIT3, ATF4, PERK, GSH, ROS, Calpains	CHAC1 upregulation is linked to GSH depletion and ROS overproduction in the lens. The UPR is activated, leading to oxidative stress, cell death, and cataract formation in Mip-mutant mice	Zhou et al. (2016)
Liver Injury						
Acetaminophen (APAP)-induced Acute Liver Injury (ALI)	AML12 (mouse hepatocytes)	*In vivo*: C57BL/6J male mice treated with APAP (500 mg/kg) and salidroside (100 mg/kg) *In vitro*: AML12 cells treated with APAP (20 mM) and salidroside (10 μM)	Salidroside inhibits ferroptosis by reducing CHAC1-mediated GSH degradation and restoring GPX4 activity	CHAC1, GPX4, PERK, eIF2α, ATF4, AMPK, SIRT1	- CHAC1 is upregulated in APAP-induced ALI and degrades GSH, contributing to ferroptosis - Salidroside inhibits CHAC1 expression via suppression of the PERK-eIF2α-ATF4 axis, reducing GSH degradation - Salidroside activates the AMPK/SIRT1 pathway, further enhancing its protective effect by inhibiting CHAC1-mediated ferroptosis	Xu et al. (2023)
Carotid Atherosclerosis (Plaque Rupture)						
Carotid Atherosclerosis (Plaque Rupture)	N/A	*In vivo*: Human carotid artery plaques from endarterectomy	Promotes apoptosis, indirectly contributing to ferroptosis	CHAC1, CHOP, ATF3, KDEL	CHAC1, as a downstream effector of CHOP, promotes apoptosis in rupture-prone plaques, contributing to plaque instability. CHAC1’s expression is localized to macrophage-rich regions of the plaque	Dorweiler et al. (2014)
Diabetes						
β-cell stress and apoptosis (Diabetes)	Min6 (mouse insulinoma cells)	*In vivo*: Primary mouse islets; *In vitro*: Min6 cells treated with Tg, palmitate	Induces apoptosis during stress, potential link to ferroptosis	CHAC1, PDX1, ATF4, ATF5, Gpt2, Slc7a1	CHAC1 is upregulated in β-cells during stress and is regulated by PDX1, ATF4, and ATF5. CHAC1 contributes to β-cell apoptosis by degrading GSH(GSH), an important process that can lead to ferroptosis	Juliana et al. (2018)
Endometriosis (EMS)						
Endometriosis (EMS)	Ectopic Endometrial Stromal Cells (EESCs) and Normal Endometrial Stromal Cells (NESCs)	- *In vitro*: Primary isolated cells from EMS patients - *In vivo*: EMS mouse model with endometrial implantation cysts	- Resveratrol promoted ferroptosis in EESCs by increasing oxidative stress - Decreased levels of GSH(GSH), increased Malondialdehyde (MDA), and lipid ROS levels indicated ferroptosis induction	- miR-21-3p - p53 - SLC7A11 - Ptgs2 (Prostaglandin-endoperoxide synthase 2) - CHAC1 (GSH-specific gamma-glutamylcyclotransferase 1)	- CHAC1: Acts as a marker of ferroptosis and promotes ferroptosis by depleting GSH, leading to an accumulation of ROS in cells - Resveratrol increased CHAC1 expression in EESCs, which facilitated ferroptosis - Knockdown of p53 reversed resveratrol’s effect on ferroptosis, but resveratrol restored ferroptosis in the presence of CHAC1 expression - CHAC1, alongside Ptgs2, was crucial in mediating ferroptosis-related therapeutic effects of resveratrol in both *in vitro* and *in vivo* EMS models	Zou et al. (2024)
Cachexia						
Cancer Cachexia	Pan02 (Pancreatic Cancer), C26, TOV21G (Ovarian Cancer), HT-1080 (Fibrosarcoma)	*In vivo*: Pan02 cancer cachexia model (mice) *In vitro*: HEK-293T	No significant prevention of muscle wasting despite increased GSHlevels	CHAC1, GSH, GPX4	CHAC1 expression increases in muscle wasting conditions (e.g., fasting, cancer cachexia) and reduces GSHlevels, promoting oxidative stress. Inactivation of CHAC1 preserves GSHbut does not prevent muscle wasting	Li et al. (2023)

## 6 CHAC1 in malignancies

**FIGURE 10:**
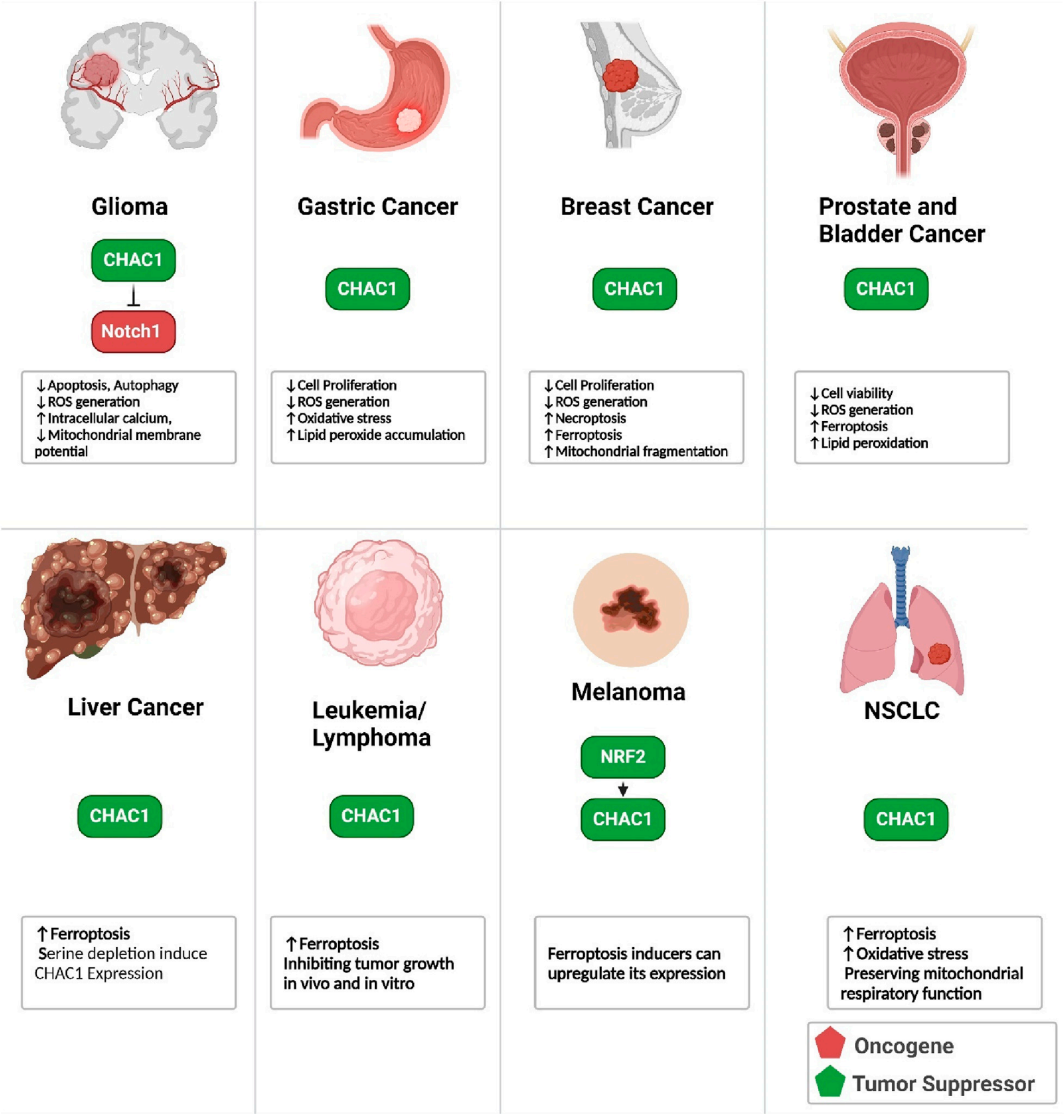
Role of CHAC1 in different cancers.

CHAC1 exhibits significant influence in the context of tumor development and progression. It contributes to the induction of ER stress. It is involved in the mechanisms of ferroptosis, necroptosis, and apoptosis across various cancer types, including metastatic melanoma, breast, prostate, and liver cancers, as well as Burkitt’s lymphoma and glioblastoma multiforme. Additionally, overexpression of CHAC1 can enhance the sensitivity of tumor cells to chemotherapy by reducing intracellular GSH levels, thereby increasing oxidative stress and promoting cell death. This multifaceted role of CHAC1 underscores its potential utility as both a diagnostic marker and a therapeutic target in oncology (Zhang et al., 2024). In this chapter, we review the role of CHAC1 in each malignancy (Figure 10).

### 6.1 Glioma

Glioma is a prevalent and aggressive type of central nervous system tumor, notorious for its low survival rates. According to the World Health Organization (WHO), gliomas are categorized into four histopathological grades, with the higher grades (III-IV) being malignant and accounting for about 60% of glioma cases (Tabnak et al., 2021b). CHAC1 significantly functions in glioblastoma-initiating cells (BTICs) by promoting apoptosis and inhibiting NOTCH signaling. Research has identified CHAC1 as a direct target of the FOXG1 and TLE1 transcriptional repression complex, which reduces CHAC1 expression in glioblastoma (GBM). Increased CHAC1 levels, triggered by treatments like Temozolomide (TMZ), facilitate apoptotic processes and improve the effectiveness of anti-glioma therapies. Additionally, CHAC1 hampers the activation of NOTCH3, thus diminishing NOTCH-mediated oncogenic pathways that are essential for GBM advancement. By repressing CHAC1, the FOXG1:TLE1 complex aids in the survival and proliferation of GBM cells, making CHAC1 a promising target for therapeutic strategies aimed at combating gliomagenesis (Dali et al., 2018). CHAC1, upregulated by TMZ via the JNK1/c-JUN signaling pathway, significantly enhances glioma cell death through mechanisms involving apoptosis, autophagy, ROS generation, increased intracellular calcium, and mitochondrial membrane potential loss. Additionally, CHAC1 inhibits the Notch3 signaling pathway by binding to Notch3 and preventing its activation, which further contributes to TMZ’s cytotoxic effects on glioma cells. The findings suggest that targeting the CHAC1-Notch3 axis could provide new therapeutic strategies for treating glioblastoma (Chen P.-H. et al., 2017).

### 6.2 Gastric cancer

Gastric cancer is a major contributor to cancer-related mortality worldwide and is typically identified in advanced stages due to its initially asymptomatic progression. The primary forms include true gastric adenocarcinomas and gastro-esophageal junction adenocarcinomas. Significant risk factors encompass *Helicobacter pylori* infection, tobacco use, inadequate nutrition, and genetic factors. Oxidative stress significantly contributes to the development and advancement of gastric cancer by causing DNA damage and impacting various signaling pathways. Although treatment strategies have improved, the survival rate remains low, highlighting the urgent need for further research to understand the disease’s underlying mechanisms better and to develop more effective treatment options (Liu et al., 2023b). CHAC1 plays a crucial role in regulating ferroptosis within GC. The activation of CHAC1 leads to GSH depletion, thereby increasing oxidative stress and lipid peroxide accumulation, hallmarks of ferroptosis. The ATF4/CHAC1 pathway is central to this process, with ATF4 upregulating CHAC1 in response to stress signals, thus promoting ferroptosis. In GC cells, CPEB1 suppresses twist1, a negative regulator of ATF4, enhancing this pathway. Increased CHAC1 levels induce ferroptosis and inhibit GC cell proliferation and survival, indicating that targeting the ATF4/CHAC1 axis could be a promising strategy for ferroptosis-based cancer therapy. Consequently, CHAC1 serves as a key mediator in ferroptosis, aiding in the inhibition of gastric cancer progression (Wang J. et al., 2021). Ophiopogonin B (OP-B) significantly influences ChaC1 in gastric cancer by increasing its mRNA levels. ChaC1 mRNA levels were markedly elevated in gastric cancer tissues treated with OP-B compared to adjacent normal tissues. This upregulation of ChaC1 suggests that OP-B induces ferroptosis in gastric cancer cells, as ChaC1 is a marker of this form of cell death. By enhancing the expression of ChaC1, OP-B disrupts the cellular redox balance, promoting oxidative stress and leading to cancer cell death. This mechanism underscores the potential of OP-B as a therapeutic agent targeting ferroptosis in gastric cancer (Zhang et al., 2022).

### 6.3 Breast cancer

Breast cancer is a malignant tumor that originates from the cells of the breast, characterized by uncontrolled cell growth that can spread to other parts of the body. It is a leading cause of cancer-related deaths among women worldwide. One crucial aspect of breast cancer progression involves ER stress. In breast cancer, internal and external stresses can disrupt ER function, leading to ER stress and the activation of the UPR. This response aims to restore normal function but, if chronic, can contribute to cancer cell survival, growth, metastasis, and resistance to therapy. Therefore, targeting ER stress and its signaling pathways presents a potential therapeutic strategy for combating breast cancer (Xu D. et al., 2022). Researchers found that cystine starvation induces necroptosis and ferroptosis in TNBC cells, marked by increased mitochondrial fragmentation, dysfunction, and ROS production. The key molecular pathway identified involves the activation of the general control nonderepressible 2 (GCN2) kinase, leading to the phosphorylation of eukaryotic initiation factor 2 alpha (eIF2α) and the upregulation of ATF4. This pathway subsequently increases the expression of CHAC1 (Chen M.-S. et al., 2017). Through a combination of metabolomics and transcriptomics, it was found that malignant breast cancer cell lines (MCF-7 and BCC) exhibit lower activity of the methionine cycle compared to non-malignant cell lines (MCF-10A). A significant finding was the elevated levels of pyroglutamic acid in malignant cells, which was linked to the overexpression of CHAC1, resulting in the production of pyroglutamic acid and cysteinyl-glycine. The increased expression of CHAC1 in cancer cells leads to a depletion of intracellular GSH, contributing to oxidative stress and promoting cancer cell survival and proliferation. Also, a potential oncogenic role of CHAC1 has been discovered, as its overexpression correlates with more advanced stages of breast cancer and poorer prognosis (Pankevičiūtė-Bukauskienė et al., 2023). CHAC1 is a downstream effector of several key signaling pathways, including the RhoA-ROCK1 and p38 MAPK pathways, which are modulated by tumor suppressors like ARHGAP6. Overexpression of ARHGAP6, which inhibits these pathways, results in increased CHAC1 expression, thereby promoting ferroptosis and inhibiting tumor growth. Conversely, CHAC1’s role in ferroptosis makes it a potential biomarker and therapeutic target, with studies indicating that its modulation could be leveraged to induce ferroptosis selectively in breast cancer cells, thereby suppressing tumor proliferation and improving patient outcomes. The relationship between CHAC1 and other ferroptosis markers, such as PTGS2 and ACSL4, further underscores its significance in the oxidative stress and cell death mechanisms pivotal to cancer progression and treatment strategies (Chen X. et al., 2024).

### 6.4 Prostate cancer

Prostate cancer (PCa) is one of the most commonly diagnosed cancers in men, particularly affecting the prostate gland which plays a role in the male reproductive system. In PCa, androgen signaling significantly influences UPR pathways, with androgens activating IRE1α-XBP1 signaling and inhibiting the PERK-eIF2α pathway. This differential regulation suggests that UPR supports PCa cell survival and growth under stress conditions, making it a potential therapeutic target. Strategies to disrupt UPR or target ER chaperones like BiP/GRP78, which are often upregulated in PCa, are being explored to enhance treatment efficacy (Storm et al., 2016). CHAC1, a proapoptotic protein involved in ER stress, shows decreased expression in prostate cancer cells compared to normal prostate epithelial cells. Overexpression of CHAC1 in prostate cancer cell lines DU145 and 22RV1 significantly reduces cell viability and GSH levels, while its knockdown increases viability in DU145 cells. CHAC1 overexpression induces ER stress markers BIP and CHOP, and promotes ferroptosis by increasing lipid peroxides and reducing GPX4 levels. Additionally, CHAC1 enhances the sensitivity of prostate cancer cells to the chemotherapy drug docetaxel (DTX). The inhibitory effect of DTX on cell viability is more pronounced with CHAC1 overexpression and is mitigated by ferroptosis inhibitors. Thus, CHAC1’s role in inducing ER stress and ferroptosis highlights its potential as a therapeutic target to increase the efficacy of DTX in treating castration-resistant prostate cancer (He et al., 2021). CHAC1 is targeted by miR-432-5p derived from cancer-associated fibroblast (CAF) exosomes. The suppression of CHAC1 by miR-432-5p leads to decreased GSH consumption and reduced lipid ROS accumulation, thereby inhibiting ferroptosis. Overexpression of CHAC1 in prostate cancer cells increased intracellular lipid peroxidation and decreased levels of GPX4, promoting ferroptosis and enhancing sensitivity to docetaxel. Conversely, the downregulation of CHAC1 by CAF-secreted miR-432-5p decreases ferroptosis, contributing to chemoresistance. *In vivo* experiments confirmed that knocking down miR-432-5p in CAFs enhanced CHAC1 expression, increased ferroptosis, and improved the efficacy of docetaxel in reducing tumor growth. These findings suggest that targeting the miR-432-5p/CHAC1 pathway could be a potential therapeutic strategy to overcome chemoresistance in prostate cancer (Zhao et al., 2024).

### 6.5 Bladder cancer

CHAC1 is crucial in inducing ferroptosis in bladder cancer (Bca) cells. It degrades reduced GSH, an important antioxidant, into cysteinylglycine and 5-oxoproline, thereby decreasing the cellular antioxidant defense and increasing oxidative stress. High levels of CHAC1 are found in various cancers and are linked to poor prognosis. In Bca cells treated with brusatol, CHAC1 expression significantly rises, leading to GSH depletion, ROS buildup, and increased lipid peroxidation, which are key features of ferroptosis. Reducing CHAC1 expression in these cells improves cell viability, lowers ROS levels, and restores mitochondrial integrity, indicating CHAC1’s essential role in ferroptotic cell death. Additionally, CHAC1 downregulation results in increased levels of ferroptosis inhibitors such as GPX4 and SLC7A11, underscoring its pivotal role in controlling ferroptosis and its potential as a therapeutic target in bladder cancer (Yu et al., 2024).

### 6.6 Liver cancer

Hepatocellular carcinoma (HCC) is a prevalent and deadly form of liver cancer with a high incidence of recurrence and metastasis. The ER stress response plays a crucial role in the pathogenesis and progression of HCC. ERS occurs when the folding capacity of the ER is overwhelmed, leading to an accumulation of misfolded or unfolded proteins. This triggers the UPR, which can promote cell survival or apoptosis depending on the severity and duration of the stress. In HCC, ERS is implicated in tumor cell proliferation, migration, invasion, angiogenesis, and resistance to chemotherapy. Factors such as viral hepatitis, cirrhosis, and steatohepatitis can induce ERS, contributing to the transformation of normal hepatocytes into cancerous cells (Wu et al., 2021). The study by Hamano et al. reveals that extracellular L-serine depletion in Hepa1-6 hepatoma cells activates the Atf4-mediated transcriptional program, inducing genes like ChaC1. This activation is transient, peaking at 2 h and returning to baseline by 24 h. The addition of glycine, similar to serine, suppresses this upregulation, indicating that glycine can compensate for serine deficiency. Intracellular L-serine levels initially remain stable but significantly increase after 24 h of serine depletion, while intracellular and extracellular glycine levels decrease but recover after 24 h. Gene expression analysis shows that Phgdh and Shmt2, involved in serine synthesis, are upregulated under serine depletion. Suppressing Atf4 reduces ChaC1 expression, confirming Atf4’s role in this response. Additionally, serine or glycine supplementation mitigates cell proliferation arrest caused by serine depletion, highlighting the importance of these amino acids in cell growth and survival. These findings suggest a regulatory mechanism in Hepa1-6 cells that balances serine and glycine levels to maintain cellular function under nutrient stress (Hamano et al., 2020). Dihydroartemisinin (DHA) effectively induces ferroptosis in primary liver cancer (PLC) cells through mechanisms that are independent of the p53 status of the cells. DHA treatment led to classic ferroptosis characteristics, such as increased lipid ROS, MDA levels, iron overload, and decreased GSH synthesis and GPX4 expression. Notably, DHA activated all three branches of the UPR pathways, PERK/eIF2α/ATF4, IRE1α/XBP1, and ATF6. Knocking down key UPR transcription factors (ATF4, XBP1, or ATF6) significantly reduces DHA-induced ferroptosis, suggesting that UPR activation is crucial for this process. A key finding was the upregulation of CHAC1 expression by DHA, which was mediated through the UPR pathways. CHAC1’s promoter activity is enhanced by DHA. The upregulation of CHAC1 is critical for the DHA-induced depletion of GSH, contributing significantly to the ferroptosis observed in PLC cells (Wang Z. et al., 2021).

### 6.7 Burkitt’s lymphoma

Burkitt’s Lymphoma (BL) is an aggressive form of non-Hodgkin lymphoma that primarily affects children and young adults, with limited treatment options for older patients. Recent studies have identified artesunate, traditionally an antimalarial drug, as a potential therapeutic agent for BL due to its ability to trigger ferroptosis, a specialized form of cell death. Artesunate activates the ATF4-CHOP-CHAC1 signaling pathway, which significantly enhances ferroptosis in BL cells. CHAC1, a critical component of this pathway, is instrumental in regulating this cell death process, effectively inhibiting tumor growth in both laboratory and animal models. These findings offer promising insights into developing new treatments for BL by targeting the ATF4-CHOP-CHAC1 pathway (Wang et al., 2019).

### 6.8 Acute lymphoblastic leukemia

T cell acute lymphoblastic leukemia (T-ALL) is a type of aggressive cancer originating from T cell lineage-committed lymphoblasts, which commonly affects the bone marrow and peripheral blood. Genetic alterations in T-ALL lead to the activation of oncogenic transcription factors and the NOTCH1 signaling pathway, contributing to the disease’s progression. CHAC1, a key regulator in T-ALL, is a proapoptotic protein induced by ER stress that functions as a γ-glutamyl cyclotransferase. It interferes with the maturation of the NOTCH1 receptor by blocking its S1 cleavage, a crucial step in NOTCH1 activation. This action of CHAC1, known as the “blocker of NOTCH,” effectively reduces the cell surface expression of the full-length NOTCH1 receptor, thus inhibiting the NOTCH1 signaling pathway and contributing to the suppression of T-ALL cell viability (Chang Soo et al., 2023).

### 6.9 Melanoma

Melanoma is a type of skin cancer that arises from the melanocytes, the cells responsible for producing melanin, the pigment that gives skin its color. It is known for its aggressive nature and potential to spread to other body parts. CHAC1 plays a significant role in mediating cell death in melanoma by acting downstream of GCDH (glutaryl-CoA dehydrogenase) inhibition. When GCDH is knocked down in melanoma cells, it leads to increased glutarylation of NRF2, a transcription factor that becomes stabilized and transcriptionally active. This stabilization upregulates the expression of ATF4 and ATF3, which are crucial for the apoptotic response. CHAC1, a gene induced by ATF4 and ATF3, contributes to the execution of programmed cell death. The upregulation of CHAC1, along with other apoptotic genes like DDIT3, results in significant melanoma cell death. Consequently, CHAC1 acts as a critical mediator in the apoptosis pathway triggered by metabolic stress due to GCDH inhibition, highlighting its importance in the potential therapeutic targeting of melanoma (Verma et al., 2022). CHAC1’s expression is rapidly upregulated upon ferroptosis induction by agents such as erastin. Notably, this upregulation occurs independently of ER stress pathways, as evidenced by the lack of activation of typical ER stress markers such as ATF4, ATF6, XBP1, and CHOP during ferroptosis. Instead, CHAC1 expression is regulated by the oxidative stress response transcription factor NRF2. Upon ferroptosis induction, NRF2 activation leads to increased CHAC1 expression, contributing to the degradation of GSH and facilitating ferroptotic cell death. This positions CHAC1 as a key mediator in the ferroptotic pathway, linking oxidative stress responses to the execution of ferroptosis in melanoma cells (Gagliardi et al., 2019).

### 6.10 NSCLC

Non-small cell lung cancer (NSCLC) is the predominant form of lung cancer, representing approximately 85% of cases. It includes various subtypes such as adenocarcinoma, squamous cell carcinoma, and large cell carcinoma. NSCLC generally progresses and metastasizes more slowly compared to small cell lung cancer (SCLC) (Tabnak et al., 2021a). In NSCLC, CHAC1 plays a crucial role in preserving mitochondrial respiratory function under conditions of cysteine starvation. CHAC1, a component of the intracellular GSH cleavage system, is significantly upregulated in response to cysteine deprivation. It localizes partially to the mitochondria where it catabolizes GSH, thereby mobilizing cysteine to support the mitochondrial cysteine pool. This process is essential for maintaining the synthesis of iron-sulfur (Fe-S) clusters, which are critical for the function of several mitochondrial enzymes involved in oxidative metabolism (Shen et al., 2024). By sustaining Fe-S cluster synthesis, CHAC1 helps retain mitochondrial respiratory function and redox balance despite the absence of extracellular cystine. This metabolic adaptation, while crucial for mitochondrial function, also promotes ferroptosis in NSCLC cells under cysteine-limiting conditions, linking CHAC1 activity to cell death pathways. Consequently, CHAC1’s function in NSCLC highlights its dual role in supporting cellular metabolism and contributing to the vulnerability of cancer cells to ferroptosis (Ward et al., 2024).

### 6.11 Colon cancer

Colitis-associated cancer (CAC) is a type of colorectal cancer that develops from prolonged inflammation in the colon, typically associated with inflammatory bowel disease (IBD). This cancer is highly lethal, with approximately 10%–20% of IBD patients developing CAC within 30 years, and about half of these patients dying from the condition. The chronic inflammation underlying CAC drives cancer development through oxidative stress, which causes genomic instability and compromises cell viability. CHAC1 plays a crucial role in the pathogenesis of CAC by promoting the breakdown of GSH. Elevated levels of CHAC1, often induced by ER stress, lead to reduced GSH levels, increased oxidative stress, and greater vulnerability to cell death processes like ferroptosis. This pathway is integral to the onset and progression of CAC, making CHAC1 a key focus for understanding and potentially treating the disease (Zhang et al., 2021).

### 6.12 CHAC1 in cancer prognosis

Although increased CHAC1 activity is associated with ferroptosis induction, we can see that upregulation of CHAC1 is commonly associated with poor prognosis in cancer patients. Increased expression of CHAC1 in cancer cells is linked to poor prognosis despite its role in degrading GSH and promoting ferroptosis. While GSH is essential for inhibiting ferroptosis by neutralizing ROS, CHAC1 degrades GSH, reducing the cell’s capacity to prevent oxidative damage. However, cancer cells often adapt to oxidative stress by upregulating CHAC1, which can help them manage and exploit oxidative environments, leading to increased resilience and survival. This adaptation can also contribute to resistance against therapies that induce oxidative stress or ferroptosis, supporting tumor growth and complicating treatment outcomes. Thus, CHAC1’s upregulation enhances the survival and aggressiveness of cancer cells in the tumor microenvironment, leading to a poor prognosis (Parande et al., 2024). Table 3 shows the prognostic value of CHAC1 in different cancers.

**TABLE 3:** Prognostic and clinicopathological value of CHAC1 across different cancers.

Type of cancer	Prognostic ratio	Clinicopathological findings	Other highlights
Breast Cancer (Goebel et al., 2012)	Transcript variant 1: RRdeath 6.7 (2.4–18.9); RRrelapse 6.7 (2.1–21.3); Transcript variant 2: RRdeath 4.9 (2.0–12.4); RRrelapse 8.0 (2.4–26.8)	Higher CHAC1 mRNA expression in poorly differentiated tumors (*P* = 0.004), hormone receptor-negative tumors (*P* < 0.001), and larger tumor size (*P* = 0.011)	CHAC1 knockdown suppressed cell migration, while overexpression increased it
Breast Cancer (Mehta et al., 2022a)	High CHAC1 expression correlates with poor overall survival (OS), distant metastasis-free survival (DMFS), and recurrence-free survival (RFS)	High CHAC1 in tumor tissues, aggressive subtypes like HER2 and TNBC, associated with advanced stages and lymph node metastasis	CHAC1 expression linked to mutant p53, hypomethylation of CHAC1 promoter
Breast Cancer (Jahn et al., 2017)	High CHAC1 expression associated with HR = 2.49	Higher CHAC1 levels linked to advanced tumor stage, lymph node positivity, and higher degree of malignancy	Addition of CHAC1 to prognostic score resulted in 16% reclassification, significantly improving treatment stratification
Ovarian Cancer (Goebel et al., 2012)	Younger patients (<62.6 years): Poorer RFS (*P* = 0.030) and OS (*P* = 0.012)	Higher CHAC1 mRNA expression in poorly differentiated tumors (*P* = 0.024) and advanced-stage cancers (*P* = 0.026)	CHAC1 knockdown suppressed cell migration, while overexpression increased it
Triple-negative breast cancer (TNBC) (Mehta et al., 2022b)	High CHAC1 expression correlates with poor prognosis	Associated with high Ki67 index, indicating high proliferation rate	CHAC1 expression significantly higher in lymph node-positive tumors
Uveal Melanoma (Liu et al., 2019)	High CHAC1 expression correlated with poor prognosis and was an independent predictor for UM patients	Higher CHAC1 levels correlated with liver metastases, histological type, pathological-M, recurrence, and overall survival	CHAC1 knockdown inhibited cell proliferation and migration; PI3K/AKT signaling pathway was suppressed in CHAC1-silenced cells
Kidney Renal Clear Cell Carcinoma (KIRC) (Li D. et al., 2021)	Upregulated CHAC1 is an independent risk factor for poor prognosis (*P* = 0.014)	CHAC1 expression correlated with higher T stages, grades, and stages; associated with immune pathway markers and checkpoint genes	CHAC1 influences cell death in KIRC cell lines but has limited impact on cell migration and invasion; associated with ferroptosis and GSH metabolism abnormalities
Stomach Adenocarcinoma (STAD) (Xiao et al., 2022)	High-risk group had significantly lower survival probabilities. ROC curve AUCs for 1, 3, and 5 years were 0.67, 0.7, and 0.75 respectively	Tumor staging and ferroptosis-related prognostic signature were significantly correlated with overall survival. CHAC1 was downregulated, NOX4 was upregulated in STAD tissues compared to normal tissues	Constructed a nomogram to predict 1-, 2-, and 3-year survival probabilities. High-risk group had higher stromal score, immune score, and ESTIMATE score. Significant differences in immune cell infiltration between high and low-risk groups, with high-risk group showing increased M2 macrophages, regulatory T cells (Tregs), and follicular helper T cells (TFH)

## 7 CHAC1 in cancer treatment

CHAC1 has gained recognition as a critical factor in the evolving field of cancer treatment, presenting new opportunities to enhance therapeutic outcomes. This section explores two pivotal concepts: regulating chemo-Immuno therapy effects and pharmacotherapy, highlighting the integral role of CHAC1. Enhancing our understanding and control of chemotherapy response paves the way for more effective, individualized cancer therapies. In contrast, pharmacotherapy involves the application of pharmaceutical agents to combat cancer, focusing on maximizing drug effectiveness while curbing resistance (Zhang et al., 2024). Investigating CHAC1’s role in these areas promises to reveal innovative approaches that could transform cancer treatment, ultimately improving survival rates and the quality of life for patients.

### 7.1 CHAC1 in response to chemotherapy

CHAC1 upregulation is frequently seen in tumors following receiving chemotherapy or immunotherapy. CHAC1 plays a significant role in enhancing chemotherapy sensitivity in prostate cancer cells by inducing ER stress and promoting ferroptosis. Overexpression of CHAC1 decreases cell viability and intracellular GSH levels, leading to increased sensitivity to the chemotherapeutic agent docetaxel (DTX). This sensitivity is associated with upregulated ER stress markers (BIP and CHOP) and higher intracellular lipid peroxide levels, both of which contribute to ferroptosis (Zhao et al., 2024; He et al., 2021). Nelfinavir is an HIV protease inhibitor that has been repurposed as an anti-cancer drug. It exerts its anti-cancer effects primarily through the induction of ER stress and the inhibition of the AKT/mTOR pathway. In the context of T-cell acute lymphoblastic leukemia (T-ALL), nelfinavir has been shown to inhibit the NOTCH1 pathway by blocking γ-secretase activity through the inhibition of presenilin processing. Additionally, nelfinavir upregulates the expression of CHAC1, a proapoptotic protein and negative regulator of the NOTCH pathway. CHAC1 interferes with NOTCH1 maturation by blocking S1 cleavage, thereby inhibiting NOTCH signaling. This multifaceted mechanism highlights nelfinavir’s potential as a therapeutic agent against T-ALL by targeting both NOTCH1 and mTOR pathways (Chang Soo et al., 2023). Similarly, temozolomide (TMZ) significantly upregulates CHAC1 expression in glioma cells, a process mediated through the JNK1/c-JUN signaling pathway. This upregulation occurs in both dose- and time-dependent manners, with CHAC1 mRNA and protein levels showing substantial increases shortly after TMZ treatment. The elevated levels of CHAC1 enhance TMZ-mediated cytotoxic effects, leading to increased apoptosis in glioma cells. Specifically, overexpression of CHAC1 results in heightened activation of apoptotic markers such as caspase-3 and PARP degradation. Furthermore, CHAC1 upregulation contributes to the induction of autophagy, ROS generation, increased intracellular calcium levels, and loss of mitochondrial membrane potential, all of which collectively promote cell death. Therefore, CHAC1 plays a crucial role in enhancing the cytotoxic efficacy of TMZ in glioma therapy (Chen P.-H. et al., 2017). Bortezomib is a proteasome inhibitor used as a first-line treatment for multiple myeloma, a type of blood cancer characterized by excessive proteasome activity. While bortezomib significantly improves patient survival, resistance to the drug often develops, primarily due to increased proteasome activity and altered cellular signaling pathways. Recent research has shown that pretreatment with omega-3 fatty acids DHA and EPA can overcome bortezomib resistance. This is achieved by promoting the degradation of GSH, a key antioxidant in cells. The pretreatment decreases GSH levels, heightening oxidative stress and enhancing the cytotoxicity of bortezomib. The mechanism involves the activation of the NRF2-ATF3/4-CHAC1 signaling pathway, which is crucial for GSH degradation and subsequent cancer cell apoptosis. Thus, targeting GSH metabolism through DHA/EPA pretreatment offers a promising strategy to improve bortezomib efficacy in resistant multiple myeloma cases (Chen J. et al., 2021). Arsenic trioxide (As2O3) is utilized as a chemotherapy agent, primarily for treating acute promyelocytic leukemia (APL), a subtype of acute myeloid leukemia (AML). It induces remission by degrading the PML-RARα fusion protein, which is crucial in APL pathogenesis. The drug works through mechanisms like promoting apoptosis, inhibiting angiogenesis, and disrupting cellular redox balance (Jiang et al., 2023). In HaCaT cells, the knockdown of CHAC1 using siRNA results in elevated intracellular GSH levels, which subsequently reduces the sensitivity to As(III) and hydrogen peroxide (H2O2)-induced cytotoxicity. This protective effect is abrogated by buthionine sulfoximine (BSO), an inhibitor of GSH biosynthesis, confirming the role of GSH in mitigating arsenite toxicity. Additionally, CHAC1 is implicated in the induction of apoptosis upon As(III) exposure, as evidenced by increased levels of apoptotic markers and caspase-3 cleavage in cells with intact CHAC1 expression. Thus, CHAC1 exacerbates arsenite cytotoxicity by degrading GSH and promoting apoptosis in response to arsenic exposure (Sumi et al., 2023). Checkpoint blockade therapies, such as PD-1 inhibitors, enhance the anti-tumor immune response by blocking inhibitory signals that limit T cell activity. Tumor CHAC1 expression is upregulated during amino acid deprivation, which sensitizes tumor cells to ferroptosis and enhances their susceptibility to cytotoxic T lymphocyte (CTL)-mediated killing. In immunotherapy, high CHAC1 levels correlate with improved patient outcomes, as it facilitates ferroptosis, leading to more effective tumor cell death and promoting robust anti-tumor immunity. Conversely, CHAC1 deficiency in tumor cells results in resistance to CTL-mediated cytotoxicity and impairs the efficacy of checkpoint blockade therapies, highlighting its pivotal role in mediating the success of immunotherapy (Xue et al., 2023). However, in a controversial study, CHAC1 was identified as a uniquely downregulated gene in rhabdomyosarcoma (RMS) tumors treated with the combination therapy of low-dose vincristine (VCR) and FOXM1 inhibitor RCM1, delivered via nanoparticles. RNA-seq analysis revealed that CHAC1 was one of the most significantly downregulated genes in the combination treatment group compared to single-agent treatments or control. qRT-PCR and immunostaining confirmed decreased CHAC1 expression at both mRNA and protein levels in tumors treated with combination therapy. The knockdown of CHAC1 in RMS cells *in vitro* recapitulated the effects of the combination therapy, leading to reduced cell proliferation, mitosis, and increased apoptosis. Furthermore, high CHAC1 expression was associated with poor prognosis in sarcoma patients, indicating its potential role in RMS tumorigenesis and as a therapeutic target (Donovan et al., 2023). These data show that the correlation between CHAC1 expression and therapy response is highly dependent on the type of tumor and the therapeutic agent administered.

### 7.2 Pharmacotherapeutic targeting of CHAC1

The therapeutic targeting of CHAC1 has been extensively studied across various cancer types using a wide array of medications (summarized in Table 4). Among the different cancer types explored, gastric cancer (Tseng et al., 2021; Zhang et al., 2022; Kong et al., 2024; Chu et al., 2022; Guan et al., 2020) and head and neck cancers (Wang X. et al., 2022; Joo et al., 2012) have received the most attention. The medications investigated for CHAC1 targeting include natural products, synthetic derivatives, traditional Chinese medicine, and common pharmaceuticals like metformin. Notably, various natural extracts such as Tanshinone IIA, Ophiopogonin B, and Tremella fuciformis polysaccharides have been evaluated for their effects on CHAC1 in these cancers. The medications studied are diverse and include bacteriocins, amino acid derivatives, natural products, and synthetic compounds. These agents consistently induce the expression of CHAC1 across different cancer types, often leading to apoptosis, increased oxidative stress, and enhanced ferroptosis. For example, drugs like Nisin and Tanshinone IIA have shown increased CHAC1 expression, resulting in apoptosis and ferroptosis, respectively. Therapeutic agents targeting CHAC1 frequently affect key stress response pathways, such as the UPR and ER)stress pathways. ATF4, a transcription factor involved in the integrated stress response, emerges as a commonly affected target across many studies. Other recurrent targets include ER stress markers like DDIT3 (CHOP), GRP78, and elements of the oxidative stress response such as NRF2 and GPX4. These pathways play a critical role in mediating the cellular effects of CHAC1 induction. Following treatment with CHAC1-inducing agents, several common cellular changes are observed. Increased apoptotic cell death is a frequent outcome, as evidenced by the upregulation of markers like caspases and the BAX/Bcl-2 ratio. Additionally, there is a notable increase in ROS and related oxidative stress markers, often coupled with a depletion of intracellular GSH. This indicates that oxidative stress is a crucial mechanism in the therapeutic action of these agents. Furthermore, many medications induce ferroptosis, particularly with compounds like Dihydroartemisinin and Brusatol. Induction of ER stress and subsequent autophagy is another common cellular response, marked by increased expression of UPR components and autophagy markers such as LC3B. In conclusion, the pharmacotherapeutic targeting of CHAC1 across various cancer types reveals a consistent pattern of inducing cellular stress responses that lead to apoptosis, oxidative stress, and ferroptosis. The consistent induction of CHAC1 and its downstream effects underscore the potential for developing new cancer treatments that exploit these pathways. The observed cellular changes, particularly those involving oxidative stress and ER stress pathways, highlight CHAC1 as a promising therapeutic target in oncology.

**TABLE 4:** Therapeutic induction of CHAC1 across different cancers.

Therapeutic name	Type of medication	Cancer type	Study type	Effect on CHAC1	Other targets	Highlights
Nisin (Joo et al., 2012)	Bacteriocin and food preservative	HNSCC	*In vitro* and *In vivo*	↑	PDE4C, S100P, CYP2B6, LAMA4	↑Calcium influxes and apoptosis ↓Cell proliferation ↓Tumor volume *in vivo*
Active mixture of serum-circulating small molecules (Scheffer et al., 2020)	Amino acids, monosaccharides, nucleobases, and vitamins	Cervical Cancer	*In vitro*	↑	ATF3, ATF4, mIR-3189-3p, DDIT3 and GDF15	↑ ISR and ER stress induction ↑Cytotoxic, anti-proliferative and apoptosis inducing effects
CTet (Galluzzi et al., 2012)	Indole-3-carbinol cyclic tetrameric derivative	Breast Cancer	*In vitro*	↑	ER stress response (e.g., DDIT3/CHO, ATF3, HSPA5/BiP/GRP78, CEBPB, ASNS) and autophagy (e.g., MAP1LC3B)	↑ ER stress induction ↑ Xbp-1 Splicing ↑ Ubiquitinated Substrates ↑ Cell Cycle Arrest ↑ Autophagy
Farnesol (Joo et al., 2015)	Natural product in several related isoprenoids, including perillyl alcohol and geraniol	T Lymphoblastic Leukemia	*In vitro*	↑	ATF4-ATF3-CHOP, GRP78, and Caspase 3,9	↑Intrinsic pathway of apoptosis ↓Proliferation ↑Intracellular cation levels ↑Plasma membrane depolarization ↑MEK/ERK1/2, p38, and JNK Pathway
Tetra-O-methyl nordihydroguaiaretic acid (M4N)+ Chemotherapy (Kimura and Huang, 2016)	Natural Lignan extracted from Larrea tridentata	Pan-cancer	*In vitro*	↑	ATF4/DDIT3	↑ GSHdegradation into 5-oxoproline, cysteine (cystine), and glycine and oxidative stress Synergistic induction of anticancer effect ↑ Caspase-7 cleavage ↓ Autophagy and energy metabolism ↓Content of TCA cycle-related metabolites ↑Certain glycolysis-related metabolite
Xylitol (Tomonobu et al., 2020)	Natural product extracted from extract of Cordyceps militaris	Melanoma and pancreatic cancers	*In vitro* and *In vivo*	↑	ER stress (BiP, ATF4), oxidative stress	↑Selective cancer cell death via oxidative stress ↑ ER stress and CHAC1 induction ↑Sensitizes cancer cells to chemotherapy
Tanshinone IIA (Guan et al., 2020)	Pharmacologically active component isolated from the rhizome of Salvia miltiorrhiza Bunge (Danshen)	Gastric Cancer	*In vitro* and *In vivo*	↑	Ptgs2, SLC7A11	↑ Lipid peroxidation, ↑ p53 upregulation, ↓ xCT downregulation, ↓ intracellular GSH and cysteine levels, ↑ intracellular ROS, ↑ ferroptosis
DPP23 (Sim et al., 2020)	Synthetic chalcone derivative	Pancreatic Cancer	*In vitro*	↑	GCLC, G6PD, GSTO2, GSTA5, GSTM2, GSR, GPX3/6/8, GGT1, PGD, ATF4, NAT8B	↑ ROS generation, ↑ GSH depletion, ↑ UPR, ↑ Apoptosis
Metformin (Tseng et al., 2021)	Antidiabetic drug	Gastric Cancer	*In vitro*	↑	Loc100506691, miR-26a-5p, miR-330-5p	↓ Loc100506691, Cell cycle arrest at G2/M phase ↓Cell proliferation, colony formation, and invasion
Dihydroartemisinin (Wang Z. et al., 2021)	Artemisinin derivative	Primary Liver Cancer	*In vitro*	↑	GPX4, SLC7A11, SLC3A2, ATF4, XBP1, ATF6	↑ UPR activation (PERK/eIF2α/ATF4, IRE1α/XBP1, ATF6) ↑ CHAC1 promoter activity ↑ Ferroptosis
Docosahexaenoic acid (DHA) and Eicosapentaenoic acid (EPA) (Chen M. et al., 2021)	Omega-3 fatty acids	Multiple Myeloma	*In vitro*	↑	NRF2, ATF3, ATF4	↓ GSH level, Altered expression of metabolites and enzymes in GSH metabolism, ↑ Bortezomib chemosensitivity
Glaucocalyxin A (GLA) (Wang H. et al., 2022)	Natural extract from Rabdosia japonica	Oral squamous cell carcinoma (OSCC)	*In vitro* and *In vivo*	↑	ROS-mediated ATF4/CHOP	↑ ROS-mediated mitochondrial and ER stress-induced apoptosis ↓ Tumor growth *in vivo* ↑ CHAC1 expression associated with better survival rates in OSCC patients
Ophiopogonin B (OP-B) (Zhang et al., 2022)	Natural extract from Radix Ophiopogon japonicus	Gastric cancer	*In vitro* and *In vivo*	↑	GPX4/xCT system	↓ GPX4 and xCT expression, ↑ ferroptosis, ↓ tumor growth and weight *in vivo*
Fuzheng Nizeng Decoction (FZNZ) (Chu et al., 2022)	Traditional Chinese Medicine Decoction	Gastric precancerous lesions	*In vitro*	↑	GPX4, ATF3, CHOP	↑Ferroptosis and ER stress, increases intracellular ferrous iron, ROS, malondialdehyde ↓GPX4/GSH.
NCX4040 (Sinha et al., 2023)	Non-steroidal nitric oxide donor	Colorectal Cancer	*In vitro*	↑	GPX4, NOX4, COX2	↑ ROS formation, ↑ Lipid peroxidation, ↑ Modulation of oxidative stress and inflammatory response genes, Enhanced cytotoxicity with erastin or RSL3, Inhibited cytotoxicity with ferrostatin-1, Potential ferroptosis-mediated cell death, ↑ Energy metabolism and lipid metabolism
Tremella fuciformis polysaccharides (TFPs) (Kong et al., 2024)	Polysaccharides from edible mushroom	Epstein-Barr virus-associated gastric cancer (EBVaGC)	*In vitro*	↑	NRF2, HO-1, GPX4, xCT	TFPs induce ferroptosis in EBVaGC cells by inhibiting NRF2/HO-1 signaling ↓ Cell viability in dose- and time-dependent manner, ↑ Cell death and reduced migration, ↑ PTGS2 and Chac1 mRNA levels, Suppressed NRF2/HO-1/GPX4/xCT expression, Overexpression of NRF2 rescues TFP-induced effects
Hederagenin (Hed) (Lu et al., 2024)	Triterpenoid compound	Lung Cancer	*In vitro* and *In vivo*	↑	BAX, Bcl-2	↓Lung cancer progression by activating CHAC1-mediated ferroptosis ↑ Apoptosis ↓Proliferation, and ↑ROS, MDA, and Fe^2+^ levels
Brusatol (Yu et al., 2024)	Natural compound	Bladder Cancer	*In vitro* and *In vivo*	↑	Nrf2, SLC7A11, GPX4	↑Ferroptosis ↓Cell viability, proliferation, migration, and invasion, and ↑ROS, MDA, and Fe^2+^ levels

## 8 Conclusion

This detailed review of CHAC1 highlights its complex role in various cellular processes and its significant impact on disease pathology, particularly in cancer. CHAC1 is a key regulator of oxidative stress. By breaking down GSH, CHAC1 directly affects the redox balance within cells. This is crucial because GSH is a primary antioxidant that neutralizes ROS. The depletion of GSH by CHAC1 increases oxidative stress, leading to cellular damage and death. CHAC1 also plays a central role in ferroptosis, a type of programmed cell death driven by iron-dependent lipid peroxidation. By degrading GSH, CHAC1 hampers GPX4, an enzyme that prevents lipid peroxidation, making cells more prone to ferroptosis. This role is particularly significant in cancer cells, where ferroptosis can either promote cell death or survival depending on the context.

CHAC1 activity is linked to cancer progression, influencing redox balance, cancer cell survival, proliferation, and therapy response. Many cancers exhibit CHAC1 upregulation, which correlates with poor prognosis. This upregulation is an adaptive mechanism that cancer cells use to manage oxidative stress, supporting their survival and proliferation in a hostile environment. Understanding CHAC1 regulation and function across different cancer types can provide insights for potential therapies.

CHAC1’s molecular mechanisms involve regulation by various stress response pathways, such as ISR and UPR. These pathways activate transcription factors like ATF4, ATF3, and CHOP, which increase CHAC1 expression in response to cellular stress. This intricate regulation highlights CHAC1’s role in maintaining cellular homeostasis under stress conditions and underscores its potential as a therapeutic target.

CHAC1 exhibits a complex, dual role in cancer, functioning as both an oncogene and a tumor suppressor, depending on the tumor context. On the one hand, CHAC1 demonstrates tumor-suppressive properties by promoting apoptosis and ferroptosis in response to cellular stress, such as oxidative stress and ER stress. Through pathways like the UPR, CHAC1can induce cell death by depleting GSH and generating ROS, which makes cancer cells more susceptible to stress-induced apoptosis. This has been observed in cancers such as glioblastoma and melanoma, where ChaC1 overexpression has been linked to increased cell death and a reduction in tumor growth. In this context, CHAC1 acts as a tumor suppressor, weakening cancer cells’ defenses against stress and chemotherapeutic drugs. Conversely, in some cancers, such as gastric cancer, breast cancer, and melanoma, CHAC1 has been correlated with poor prognosis, suggesting it may also play an oncogenic role. In these tumors, CHAC1can promote tumor cell survival, proliferation, and invasion, contributing to cancer progression and drug resistance. By modulating GSH levels, CHAC1 may help cancer cells manage oxidative stress, allowing them to survive under conditions that would typically induce cell death. This oncogenic behavior underscores the complexity of CHAC1’s function, where it can shift between promoting cell death in some cancers and supporting tumor survival in others. The duality of CHAC1’s role in cancer likely depends on the specific tumor microenvironment and interactions with other signaling pathways, such as the PI3K/AKT/mTOR or NRF2 pathways.

CHAC1’s role extends beyond cancer to non-malignant diseases, including neurodegenerative disorders, cardiovascular diseases, and inflammatory conditions. Its dual role in promoting oxidative stress and ferroptosis makes it a promising target for cancer therapy. Modulating CHAC1 activity could enhance cancer cell susceptibility to ferroptosis, overcoming resistance to conventional therapies. Studies show that inducing CHAC1 expression sensitizes cancer cells to chemotherapy and immunotherapy, indicating its potential in combination treatment strategies.

Moreover, this review highlights that CHAC1 upregulation enhances cancer cell sensitivity to chemotherapeutic agents by promoting oxidative stress and cell death pathways, including apoptosis and ferroptosis. Targeting CHAC1 or pathways regulating its expression could improve the efficacy of existing cancer treatments. The therapeutic potential of CHAC1 extends beyond cancer, impacting various non-malignant diseases. Its role in oxidative stress modulation and cell death pathways underscores its broader significance in human health and disease.

Research should focus on elucidating the precise mechanisms by which CHAC1 regulates oxidative stress and ferroptosis in different cellular contexts. Developing CHAC1 as a biomarker could aid in diagnosing and prognosing cancers and other diseases. Therapeutic development could involve creating small molecule inhibitors or activators of CHAC1, depending on the context. For instance, CHAC1 inhibitors could help mitigate oxidative stress in neurodegenerative diseases, while activators could enhance cancer cell death. Clinically, modulating CHAC1 in combination with chemotherapy, radiotherapy, or immunotherapy could enhance treatment efficacy and overcome resistance in cancer patients. Personalized medicine approaches could benefit significantly from insights into CHAC1’s role in disease processes. Targeting CHAC1 therapeutically presents several potential side effects and challenges that must be carefully considered to assess its feasibility as a treatment option. One major concern is that CHAC1 plays a critical role in regulating oxidative stress, GSHdegradation, and ferroptosis, essential processes for cellular redox homeostasis. Inhibiting CHAC1 could disrupt these pathways, leading to unintended consequences such as impaired cellular stress responses and the potential exacerbation of oxidative damage in non-target tissues. Additionally, CHAC1’s role in both promoting and inhibiting apoptosis and ferroptosis in a context-dependent manner across different diseases and cancers complicates its use as a therapeutic target. For instance, while inhibiting CHAC1 may enhance cancer cell sensitivity to treatments, it could also undermine its protective role in conditions like neurodegenerative diseases where ferroptosis and oxidative stress are detrimental. Furthermore, the involvement of CHAC1 in non-malignant diseases, such as diabetes, liver injury, and respiratory disorders, adds complexity, as its therapeutic modulation could potentially worsen these conditions by disrupting GSHmetabolism and increasing oxidative stress.

In conclusion, the in-depth analysis of CHAC1 underscores its critical role in maintaining cellular redox balance and regulating cell death pathways, particularly ferroptosis. Its involvement in both malignant and non-malignant diseases makes it a versatile target for therapeutic intervention. As a cancer biologist, these insights highlight the potential of CHAC1 as a biomarker and therapeutic target, paving the way for novel strategies to manage oxidative stress-related pathologies and enhance the efficacy of cancer treatments.
